# Four Neurotoxic Insecticides Impair Partner and Host Finding in the Parasitoid *Leptopilina heterotoma* and Bioactive Doses Can Be Taken up Via the Host

**DOI:** 10.1007/s10886-025-01554-w

**Published:** 2025-01-29

**Authors:** Nils Schöfer, Nathalie Saxinger, Katrin Braumandl, Joachim Ruther

**Affiliations:** Institute of Zoology, University of Regensburg, Universitätsstraße 31, 93053 Regensburg, Germany

**Keywords:** Host finding, Hymenoptera, Insecticides, Non-target species, Sex pheromone, Sublethal effects

## Abstract

In modern agriculture, control of insect pests is achieved by using insecticides that can also have lethal and sublethal effects on beneficial non-target organisms. Here, we investigate acute toxicity and sublethal effects of four insecticides on the males’ sex pheromone response and the female host finding ability of the *Drosophila* parasitoid *Leptopilina heterotoma*. The nicotinic acetylcholine receptor antagonists acetamiprid, flupyradifurone and sulfoxaflor, as well as the acetylcholinesterase inhibitor dimethoate were applied topically as acetone solutions. Males treated with all four insecticides no longer preferred the female sex pheromone in a T-olfactometer. Duration of wing fanning, an element of the pheromone-mediated male courtship behavior, was also reduced by all four insecticides. The ability of females to orientate towards host-infested feeding substrate was not affected by acetamiprid in the tested dose range. However, treatment with dimethoate, flupyradifurone and sulfoxaflor resulted in the loss of the females’ preference for host odor. At the lowest doses interfering with olfactory abilities of *L. heterotoma* in this study (acetamiprid: 0.21 ng, dimethoate: 0.105 ng, flupyradifurone: 2.1 ng and sulfoxaflor: 0.21 ng), ≥ 90% of the wasps survive insecticide treatment. Male pheromone responses and female host finding were also disturbed in those *L. heterotoma* that had developed in *D. melanogaster* larvae reared on dimethoate-treated feeding medium at sublethal levels. Hence, doses of this insecticide sufficient to interfere with chemical orientation of *L. heterotoma* can be taken up by the parasitoid via the food chain.

**Supplementary Information:**

The online version contains supplementary material available at 10.1007/s10886-025-01554-w.

## Introduction

Controlling insect pests by the use of insecticides is an important method of securing food resources but has become more controversial in recent years due to negative sublethal effects of insecticides on non-target organisms (Demirci and Gungordu 2020; Desneux et al. 2007; Müller 2018; Pisa et al. 2015). Insecticides are popular because of their ease-of-use and are deployed with various methods like spraying or seed coating. Many commercial insecticides contain active ingredients that inhibit proper central nervous system function, with many classes affecting nicotinic acetylcholine receptors (nAChR) (Nauen et al. 2015; O’Brien 1963; Simon-Delso et al. 2015; Watson et al. 2011). Amongst these substances, the neonicotinoids gained prominence as they showed high efficacy in controlling various groups of insect pests (Jeschke and Nauen 2008) and became the most popular class of insecticide on the market, at some point representing more than a quarter of global insecticide sales (Bass et al. 2015). However, after more and more adverse effects of neonicotinoids on non-target organisms, especially on bee pollinators (Decourtye and Devillers 2010; Godfray et al. 2014), became evident, the European Union (EU) first restricted and later banned the use of some neonicotinoids (European Commission 2013).

As a consequence, alternative insecticides from other classes, like butenolides and sulfoximines partly replaced neonicotinoids (Dáder et al. 2019; Gill and Chong 2021), but as they are also nAChR agonists, they are still potentially a threat for non-target organisms (Siviter and Muth 2020). Furthermore, bans on specific compounds are never global with the neonicotinoids imidacloprid, thiamethoxam and clothianidin banned in the EU but still being sold widely to other countries like China, the United States, Canada, and Brazil (Chen et al. 2019; Exactitude Consultancy 2024; Gaboardi et al. 2023). Thus, there is still a demand for studying the effects banned and alternative pesticides have on the wider environment. One alternative neonicotinoid that is still being used within the EU, as it is thought to pose a relatively low risk to bees (European Food Safety Authority 2016), is acetamiprid. Like the other neonicotinoids, acetamiprid can be applied systemically to crops, and its acute toxicity is lower when compared to other neonicotinoids (Jacob et al. 2019) though in some contexts, like foliar application, it is equally or more toxic (Horowitz et al. 1998). Another recent group of nAChR agonists is the butenolides, specifically the first commercially available active ingredient flupyradifurone (Nauen et al. 2015), which is less acutely toxic than most neonicotinoids (Bartlett et al. 2018) but is still an effective pest control agent. Similarly, sulfoxaflor, a member of the sulfoximines that is used to control populations of sap-feeding pest species (Sparks et al. 2013), is also an nAChR agonist. Another mode of action of neurotoxic pesticides is the inhibition of enzymes. Organophosphates such as dimethoate inhibit acetylcholinesterase (AChE) (Engenheiro et al. 2005), which is a vital part of proper neurofunction in insects. Dimethoate is an older insecticide (Barrett and Darnell 1967), but is still being utilized widely in the world (Ahmad et al. 2022), and hence, it is still important to study its effects.

The insects studied so far regarding sublethal effects of insecticides comprise not only pollinators and other beneficial non-target species (Agathokleous et al. 2023b; Desneux et al. 2007) but also pests (Guedes et al. 2016) and diseases vectors (Agathokleous et al. 2023a). One important group of beneficial insects are the parasitic wasps (parasitoids) that play an important role as natural enemies in natural and agricultural ecosystems (Godfray 1994; Quicke 1997). Due to their function in controlling insect pests, parasitic wasps are exposed to the same insecticides that are used against their hosts. Many species are known to feed floral and extrafloral nectar, honeydew, or guttation water (Jervis et al. 1993; Lee et al. 2004; Urbaneja-Bernat et al. 2020; Wäckers et al. 2008) which may contain various pesticide residues (Calvo-Agudo et al. 2019, 2022; Mörtl et al. 2019, 2020; Zhou et al. 2022; Zioga et al. 2020). A potential additional avenue of exposure of parasitic wasps to insecticides is via their hosts. Development of parasitoids in hosts that had been exposed to sublethal doses of insecticides is often possible but may result in decreased parasitization success and offspring number as well as indirect fitness parameters such as offspring size (Lisi et al. 2023).

As with other insects, parasitic wasps are reliant on their olfactory sense for orientation in their environment. They use semiochemicals from conspecifics, hosts, and host-damaged plants for finding mating partners and oviposition sites which is key to their reproductive success (Ruther 2013; Steidle and van Loon 2002; Turlings and Erb 2018; Wäschke et al. 2013). The processing of chemical information in the insect brain depends, among other factors, on proper nAChR function (Bohbot and Pitts 2015; Wilson and Mainen 2006). Thus, neurotoxic insecticides targeting nAChR have been shown to influence behavioral responses of parasitic wasps to olfactory cues and signals either by disrupting orientation (Delpuech et al. 1998a, b; Liu et al. 2012; Schöfer et al. 2023, 2024; Stapel et al. 2000; Tappert et al. 2017) or even stimulating it (Delpuech et al. 2005; Rafalimanana et al. 2002).

Previous research with parasitic wasps has shown that acetamiprid, dimethoate, flupyradifurone and sulfoxaflor caused interference with the olfactory orientation in the pteromalid wasps *Nasonia vitripennis* and *Lariophagus distinguendus* of the hymenopteran superfamily Chalcidoidea (Schöfer et al. 2024). These experiments revealed that all four insecticides may disrupt the response to sex pheromones and host finding in at least one of the investigated species. However, as the sensitivity of the species differed and the effects were not the same for all active substances, further species from other families need to be investigated in order to obtain a more comprehensive overview of the sublethal effects of the insecticides on the olfactory orientation of parasitoid wasps. A good candidate for this is *Leptopilina heterotoma*, a parasitic wasp of the family Figitidae and a frequently studied model system in ecology and evolution (Quicray et al. 2023). The family Figitidae belongs to the Cynipoidea which separated from the branch leading to the Chalcidoidea about 230 Mio years ago (Peters et al. 2017).

*L. heterotoma* is a solitary endoparasitoid wasp that typically parasitizes various species of the genus *Drosophila* (Papaj and Vet 1990). Hosts are found on decaying plant substrates like fermenting vegetables or fruits, where female *L. heterotoma* lay their eggs into 2nd or 3rd instar *Drosophila* larvae (Quicray et al. 2023). Parasitized larvae are able to pupate with *L. heterotoma* developing within 21–23 days (at 25 °C) inside the pupal mummies. Males emerge 1–2 days before the females. The tritrophic system feeding substrate/*D. melanogaster*/*L. heterotoma* can be well manipulated under lab conditions to study various aspects of host-parasitoid interactions (Mortimer 2013; Wertheim et al. 2003). *L. heterotoma* uses chemical information for both sexual communication and olfactory host finding. Males are attracted by a female sex pheromone consisting of (-)-iridomyrmecin, (+)-isoiridomyrmcin, two stereoisomers of iridodial, and another stereoisomer of iridomyrmecin of unknown absolute configuration (Weiss et al. 2013). The iridoids are produced in the mandibular gland (Stökl and Herzner 2016) and have also a defensive function (Stökl et al. 2012). At close range, the pheromone additionally elicits courtship behavior in males, a characteristic element of which is wing fanning (Weiss et al. 2013). High wing-fanning frequency correlates with an increased mating success of males (Lang et al. 2024). Females use fermentation products of yeasts (Dicke et al. 1984) and the aggregation pheromone of their *Drosophila* hosts (Hedlund et al. 1996) to locate host patches for oviposition. In the lab, the odor of *Drosophila-*infested rearing medium attracted *L. heterotoma* females in olfactometer bioassays (Weiss et al. 2013).

In the present study, we investigate the effects of acetamiprid, dimethoate, flupyradifurone and sulfoxaflor on (a) the attraction of *L. heterotoma* males to the female sex pheromone from a distance, (b) the male wing fanning response after contact and (c) the ability of *L. heterotoma* females to orientate towards host odor. Finally, we investigate whether dimethoate is taken up by the parasitoids in amounts impairing sexual communication and olfactory host finding when developing in insecticide-exposed hosts at sublethal levels.

## Methods and Materials

### Insects

*Drosophila melanogaster* (strain Canton S) were reared in polypropylene *Drosophila* breeding tubes in a climate-controlled cabinet at 25 °C, 50% r.h, and a 16–8 h light/dark cycle on standardized *Drosophila* feeding medium (DFM, agar, 0.7%; cornmeal, 6.4%; yeast,1.4%; soymeal, 0.8%; malt extract, 6.4%; molasses, 1.8%, nipagin, 0.2%, and water, 82.3%). Tubes were equipped with approximately 30 mixed-sex *D. melanogaster* which were allowed to mate and oviposit for two days. After this period, 20–30 mixed-sex *L. heterotoma* were added and females were allowed to parasitize the *Drosophila* larvae. At rearing conditions the next generation of *L. heterotoma* males and females emerged after approximately 21 d and 23 d, respectively. To obtain virgin and naïve wasps of defined age, parasitized host pupae were isolated after 19–20 d and transferred to individual Eppendorf tubes. Tubes were controlled daily for emerging wasps which were then used for the experiment at an age of 1–2 days (male pheromone response) and 7–10 days (female host finding), respectively.

### Insecticides

All insecticides were dissolved in HPLC-grade acetone (ROTISOLV^®^, ≥ 99.8% purity, Carl Roth GmbH, Karlsruhe, Germany) for application in bioassays. Standards of the four insecticides were purchased at analytical grade. Acetamiprid (≥ 98.0%), dimethoate (≥ 98.0%) and flupyradifurone (≥ 98.0%) standards originated from Sigma-Aldrich (Taufkirchen, Germany), Sulfoxaflor (99.23% purity) was purchased from Dr. Ehrenstorfer GmbH (Augsburg, Germany).

### Toxicity Tests

A dilution series (Table S1) was prepared of each of the insecticides in acetone, starting from a concentration of 1 mg/ml. Groups of 8 wasps (*n* = 3 replicates per dose/sex) were cooled in ice, and 210 nl of the insecticide solutions were applied topically to the abdominal tips using glass capillaries mounted to a microinjector (Nanoliter 2010, World Precision Instruments, USA). For control, wasps were treated with pure acetone. As shown previously, topical application is effective at controlling accurate dosages, and pure acetone did not show any adverse effects on survival and fitness parameters in related wasp species (Jatsch and Ruther 2021; Schöfer et al. 2023, 2024). Mortality was evaluated 72 h after application and dose-mortality curves were plotted for the four insecticides using the Quest Graph™ Lethal Dose 50 Calculator (AAT Bioquest 2023). The same tool was used to calculate the median lethal dose (LD_50_) and the mortalities of doses used in the bioassays. Except for the two highest doses of flupyradifurone, these doses caused mortalities ≤ 30% (Table 1). For the behavioral bioassays, insecticide-treated and control wasps were provided overnight with a 0.5-cm diameter filter paper disk soaked with a 50% solution of honey in water and tested the next day.

**Table 1:** Median lethal doses (LD_50_, evaluated after 72 h) and doses of the four insecticides tested in the bioassays with *Leptopilina heterotoma*. Values in brackets represent the survival percentages calculated from the functional equations of the respective sigmoidal dose-mortality curves using the online tool Quest Graph™ LD50 calculator (AAT Bioquest 2023)

	LD_50_ (ng/wasp)	Tested doses (ng/Wasp)					
Acetamiprid	2.5	0 (100%)	0.105* (97%)	0.21 (93%)	0.42 (87%)	0.525* (84%)	0.63 (81%)
Dimethoate	1.4	0 (100%)	0.053* (98%)	0.105 (96%)	0.21 (90%)	0.315* (85%)	0.42 (81%)
Flupyradifurone	31	0 (100%)	2.1* (95%)	6.3 (84%)	10.5 (76%)	15.8* (67%)	21 (60%)
Sulfoxaflor	1.2	0 (100%)	0.105* (99%)	0.21 (98%)	0.42 (90%)	0.525* (85%)	0.63 (80%)

*only tested for the investigation of the effects on courtship behavior after contact

### Production of Pheromone Extracts

Pheromone extracts for the bioassays were produced by extracting batches of females in 1.5-ml glass vials with 10 µl dichloromethane per wasp for ten minutes as described previously (Stökl et al. 2012). After removal of the females, these extracts were stored at -20 °C until needed for experimentation.

### Effects on Attraction of Males to the Female Sex Pheromone

The attraction of males to the female sex pheromone was tested in a T-olfactometer as described previously (Schöfer et al. 2023). It consisted of a T-shaped glass tube to which three Eppendorf tubes were attached, one serving as a container for the responding male and the other two for storing test and control stimuli. The T-olfactometer was set up vertically to encourage males to exit the starting tube and enter the olfactometer. One µl of the pheromone extract (representing 0.1 female equivalent) was applied to a disk of filter paper (5 mm diameter). Control disks were treated with pure dichloromethane. After evaporation of the solvent, paper disks were put into Eppendorf tubes that were attached to the two arms of the olfactometer. Tubes and olfactometer were separated by pieces of fine polyamide gauze (mesh size 125 μm). The last 5 cm of the olfactometer arms were defined as pheromone and control zone, respectively, which were separated by a 4 cm neutral zone. The Eppendorf tube containing the male was defined as the starting zone. Subsequently, males were observed for 5 min, and the time they spent in test and control zone was recorded using The Observer XT 15 scientific software (Noldus Information Technology, Wageningen, The Netherlands). The T-olfactometer was cleaned with ethanol and dried by pressurized air between uses and the arena setup was rotated by 180° after each bioassay to avoid positional bias. Polyamide gauze was replaced after each replicate.

### Effects on Wing Fanning

To assess whether the four insecticides affect the males’ courtship response (wing fanning) to the female pheromone, insecticide-treated and control males were exposed in a round glass dish (5.5 cm diameter, 1 cm height) to a 0.5 mm diameter disk of filter paper treated with 5 µl of the female extract (0.5 female equivalents). After application, the solvent was allowed to evaporate for 1 min and disks were placed in the center of the glass dish. After introduction of the males, the dish was covered with the transparent lid of a Petri dish and males were observed for 5 min. The time they spent wing fanning was recorded using The Observer XT 15 Software. Between experiments, glass dish and lid were cleaned with ethanol.

### Effects on Attraction of Females to Host Odor

To examine whether the insecticides influence the host finding ability of females, 7–10 d old *L. heterotoma* females were isolated from the breeding tubes, treated with an insecticide dose or the pure solvent (control), and transferred to an Eppendorf vial filled with 1 ml DFM and at least 5 *D. melanogaster* larvae, as experienced females are more successful in host finding (Papaj and Vet 1990). The females were conditioned overnight with the host/host substrate and tested the next day in the same T-olfactometer described above. An Eppendorf tube filled with 1 ml DFM and at least 5 *D. melanogaster* larvae was attached as stimulus to one side of the olfactometer (host zone) while the opposing Eppendorf tube remained empty (control zone). Conditioned females were transferred to empty Eppendorf tubes which were attached to the third arm of the T-olfactometer. Subsequently, the time females spent in the host and control zones were recorded for 5 min using The Observer XT 15 Software. After each replicate, the olfactometer was cleaned with ethanol and rotated by 180°, and the polyamide gauze was replaced.

### Transfer of Insecticides via the Host

To evaluate the acute toxicity of dimethoate on *D. melanogaster*, 20 larvae or 20 1-d old adults (6 replicates per concentration) were kept for 8 d on DFM treated with different concentrations of the insecticide. The treated medium was prepared by mixing 1.6 g of instant DFM per *Drosophila* rearing tube with 10 ml of distilled water and with 1 ml of various dimethoate dilutions (10000–0.1 µg/ml, Tab S2 in the Supplementary Information) dissolved in 10% acetone/water. The control DFM was prepared by adding 1 ml of 10% acetone/water instead. After the exposure time, the percentage of surviving *D. melanogaster* adults and larvae was recorded for each group. At a dimethoate concentration of 1.0 µg/ml, more than 74.2% of the *D. melanogaster* larvae survived the treatment and developed successfully to the adult stage. Therefore, this concentration and two lower ones (0.5 and 0.1 µg/ml) were chosen for the experiment. *L. heterotoma* were reared on *D. melanogaster* larvae that developed on the dimethoate-treated and solvent-treated DFM, respectively. Adult wasps having emerged from these hosts were isolated before emergence and tested in the bioassays as described above.

### Statistical Analyses

All statistical analyses were performed using PAST 4.03 software (Hammer et al. 2001). The contact pheromone data (wing fanning duration) was analyzed using a Kruskal-Wallis test following Dunn’s tests for comparing insecticide doses with the control. The number of insecticide-treated males showing wing fanning was compared to the control males by Fisher’s exact test. Both the distance pheromone data and the host finding data were analyzed by Wilcoxon matched pairs tests.

## Results

### Toxicity Tests

**Fig. 1:**
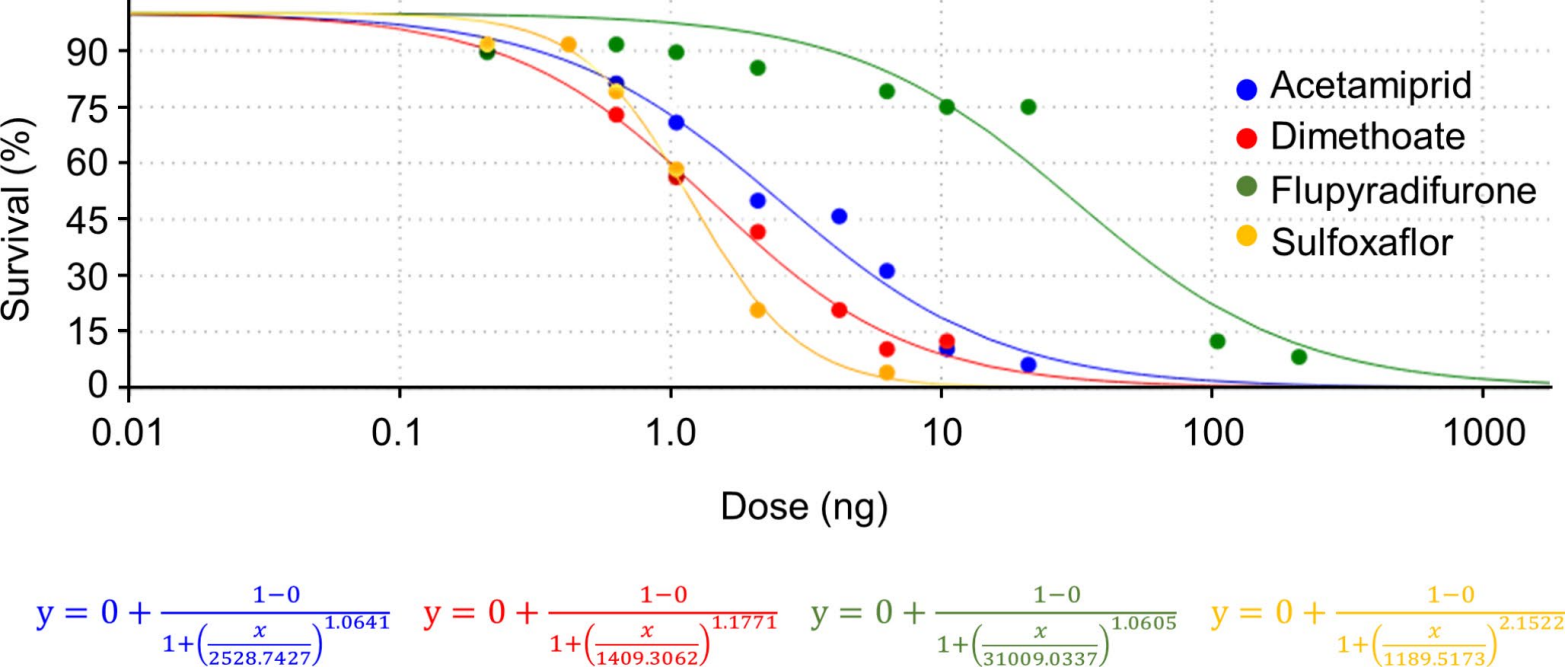
Dose-mortality curves after 72 h of *Leptopilina heterotoma* treated topically with various doses of acetamiprid, dimethoate, flupyradifurone and sulfoxaflor dissolved in acetone. Dots represent the means of the 6 groups (3 per sex) of 8 wasps treated with the same dose. Color-coded functional equations for each curve are given under the curves

The dose-mortality curves revealed large differences in the acute toxicities of the four insecticides in *L. heterotoma* (Fig. 1; Table 1). Flupyradifurone had the lowest toxicity (LD_50_ of 31 ng) followed by acetamiprid (2.5 ng), dimethoate (1.4 ng) and sulfoxaflor (1.2 ng).

### Effects on Attraction of Males to the Female Sex Pheromone

**Fig. 2:**
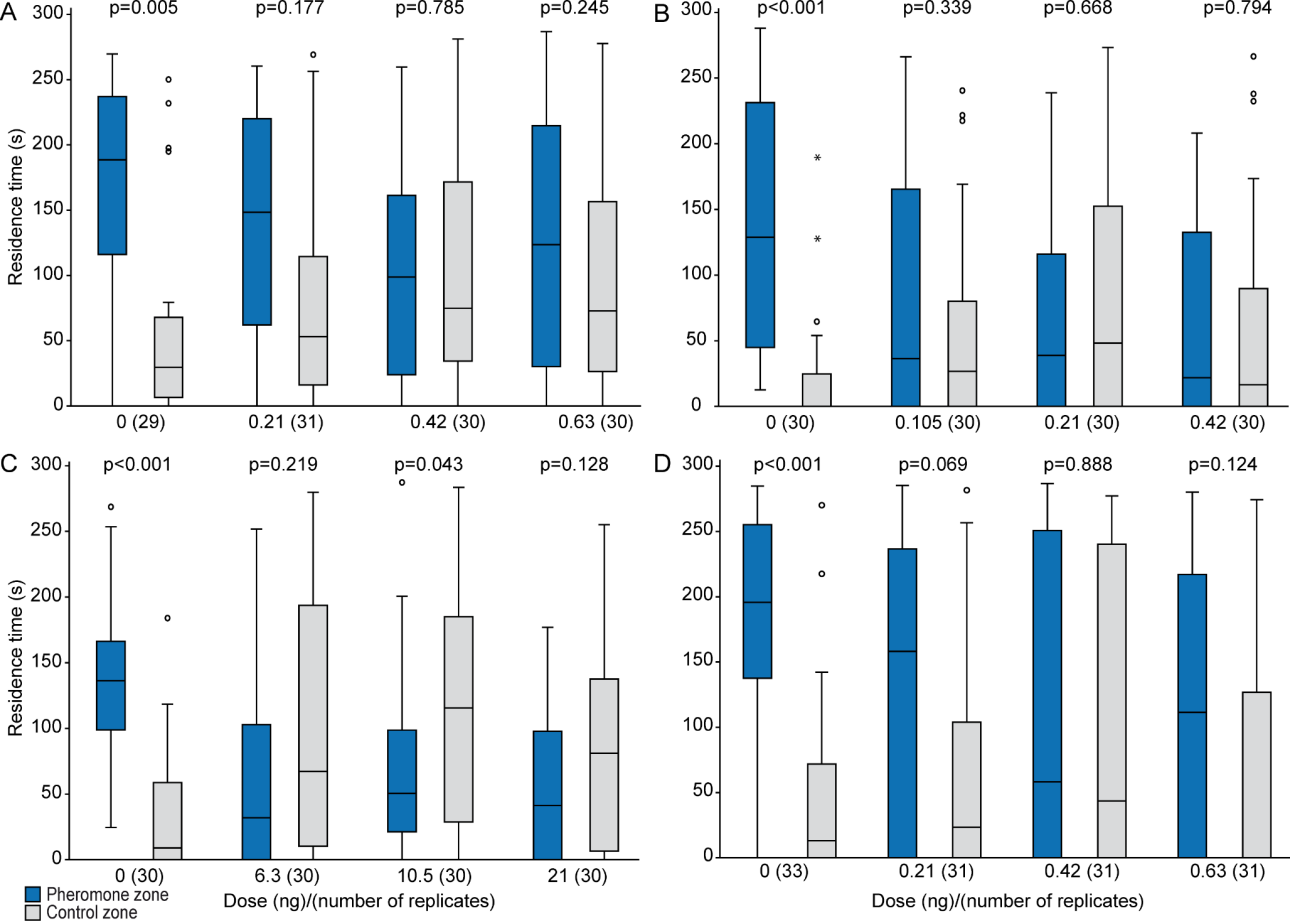
Effect of various doses of **A**. acetamiprid, **B**. dimethoate, **C**. flupyradifurone, or **D**. sulfoxaflor on the response of *Leptopilina heterotoma* males to female-derived pheromone extract in a T-olfactometer. Control males were treated with pure acetone (dose 0 ng). Shown are the residence times of males in the pheromone zone and the solvent-treated control zone of the olfactometer. Box-and-whisker plots show 25 and 75% quartiles (upper and lower end of the boxes), median (horizontal line in between), 1.5× the interquartile range (whiskers), and outliers (° means > 1.5 × and * means > 3 × interquartile range). The p-values are based on comparison between treatments and the respective control (Wilcoxon matched pairs test, number of replicates given in brackets)

In all control experiments, acetone-treated males showed significant preferences for the pheromone zone over the control zone (Fig. 2A-D, video S1 in the Supplementary Information). This preference was no longer present in insecticide-treated males at any tested dose. Males treated with the intermediate dose of flupyradifurone even preferred the control zone over the pheromone zone.

### Effects on Wing Fanning

**Fig. 3:**
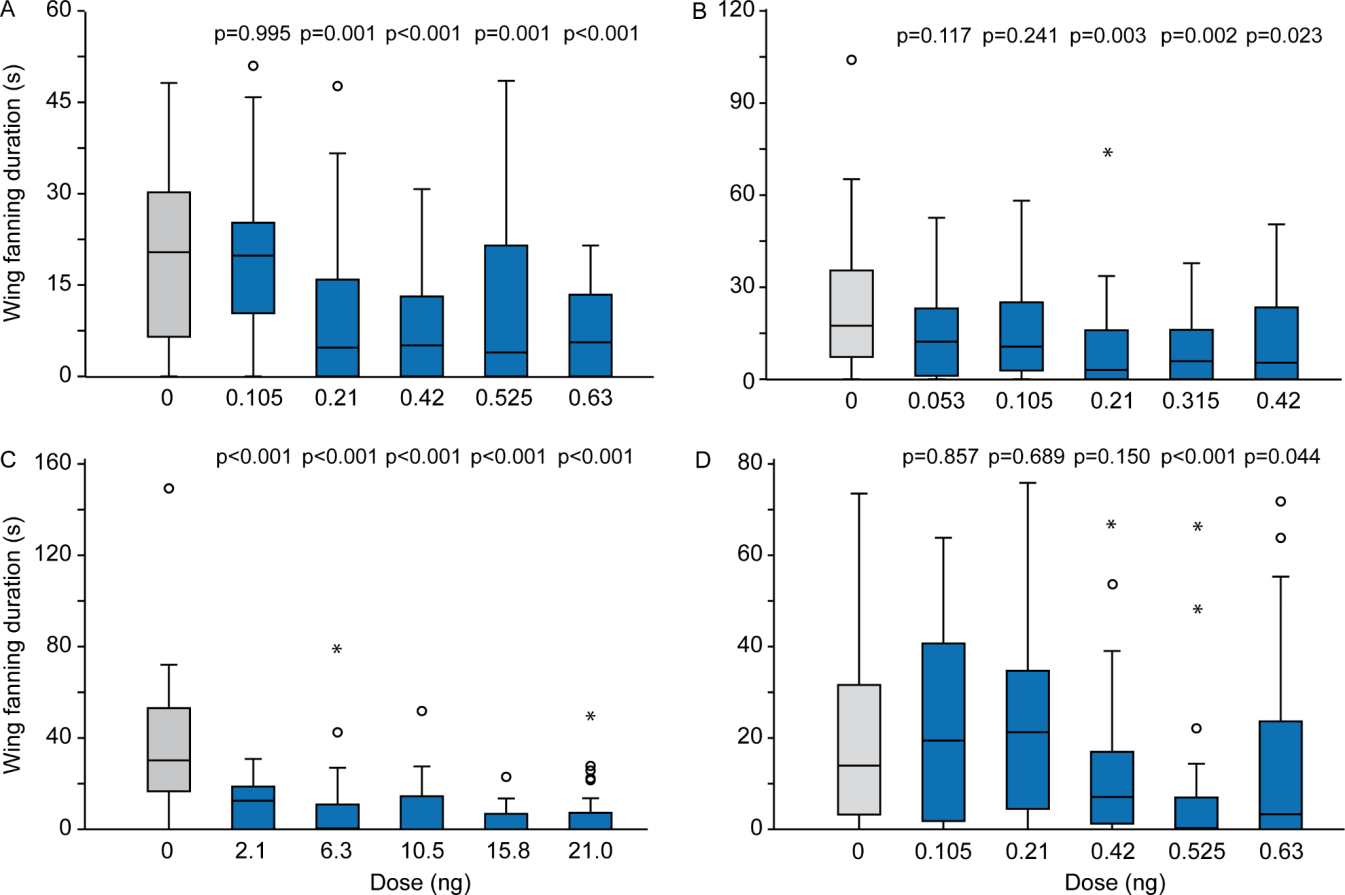
Effect of various doses (ng) of **A**. acetamiprid, **B**. dimethoate, **C**. flupyradifurone, and **D**. sulfoxaflor on the wing-fanning response of *Leptopilina heterotoma* males to female-derived pheromone extract. Shown is the total wing fanning duration within a 5 min observation period. Control males were treated with pure acetone (dose 0 ng). Box-and-whisker plots show 25 and 75% quartiles (upper and lower end of the boxes), median (horizontal line in between), 1.5× the interquartile range (whiskers), and outliers (° means > 1.5 × and * means > 3 × interquartile range). The p-values are based on comparison between treatments and the respective control (Kruskal-Wallis test and subsequent Dunn’s tests; *n* = 30)

**Fig. 4:**
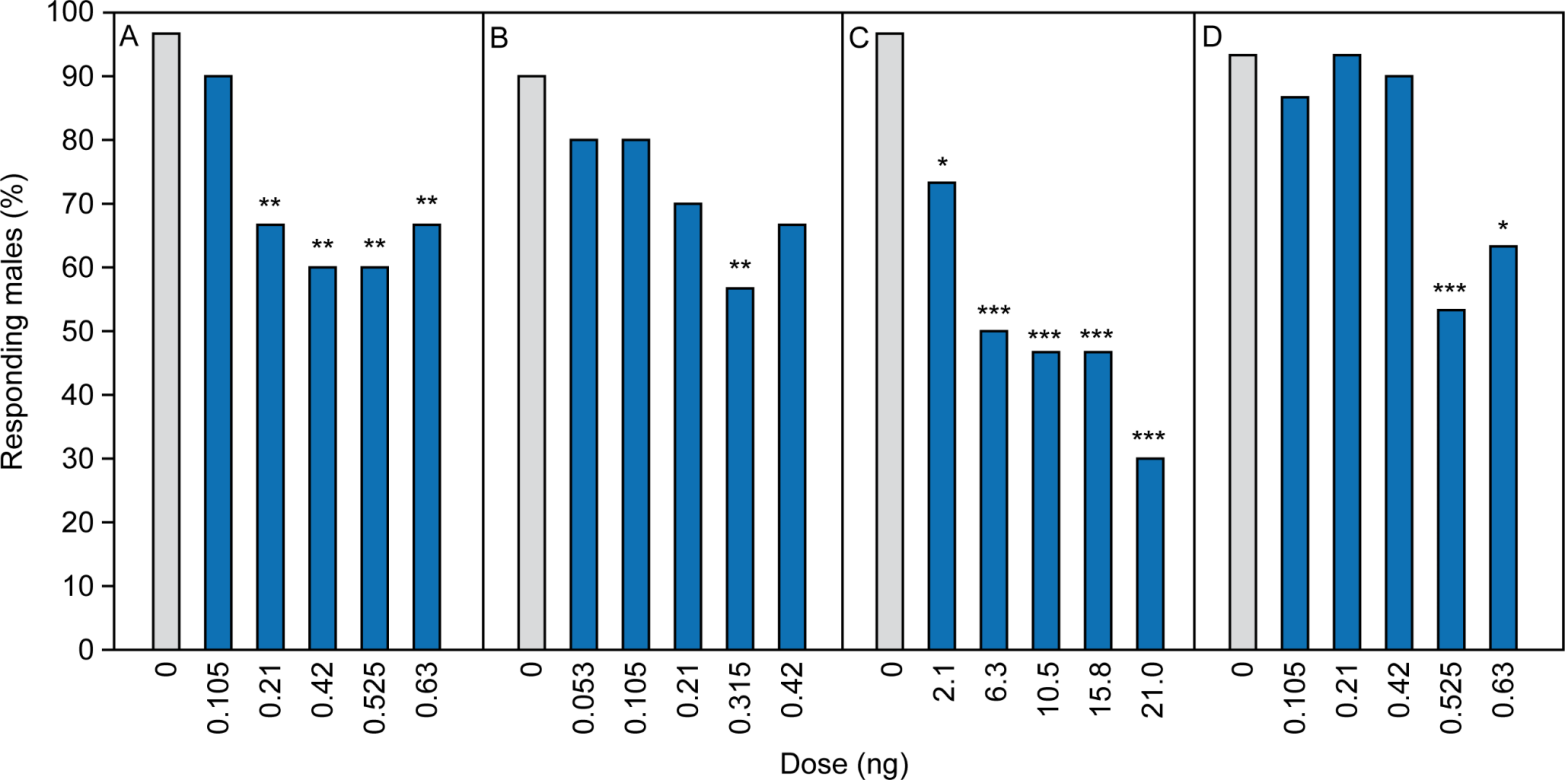
Effect of various doses (ng) of **A**. acetamiprid, **B**. dimethoate, **C**. flupyradifurone, and **D**. sulfoxaflor on the wing-fanning response of *Leptopilina heterotoma* males to female-derived pheromone extract. Shown is the percentage of males showing wing fanning. Control males were treated with pure acetone (dose 0 ng). Asterisks indicate significant differences between treatments and the respective control (Fisher’s exact test: *0.01 < *p* < 0.05, **0.001 < *p* < 0.01, ***p < 0.001; *n* = 30)

In all experiments, acetone-treated males exhibited prolonged bouts of typical wing-fanning behavior when exposed to pheromone-treated paper disks (Fig. 3A-D, video S2 in the Supplementary Information). Above a certain dose, treatment of males with all four insecticides reduced the wing fanning duration (acetamiprid: ≥ 0.21 ng, Kruskal-Wallis H = 31.71, *p* < 0.001; dimethoate: ≥ 0.21 ng, H = 13.83, *p* = 0.0151; flupyradifurone: ≥ 2.1 ng, H = 50.79, *p* < 0.001; sulfoxaflor: ≥ 0.525 ng, H = 27.02, *p* < 0.001). A significant proportion of insecticide-treated males failed to show wing fanning towards the pheromone-treated paper disks at all (Fig. 4A_D).

**Fig. 5:**
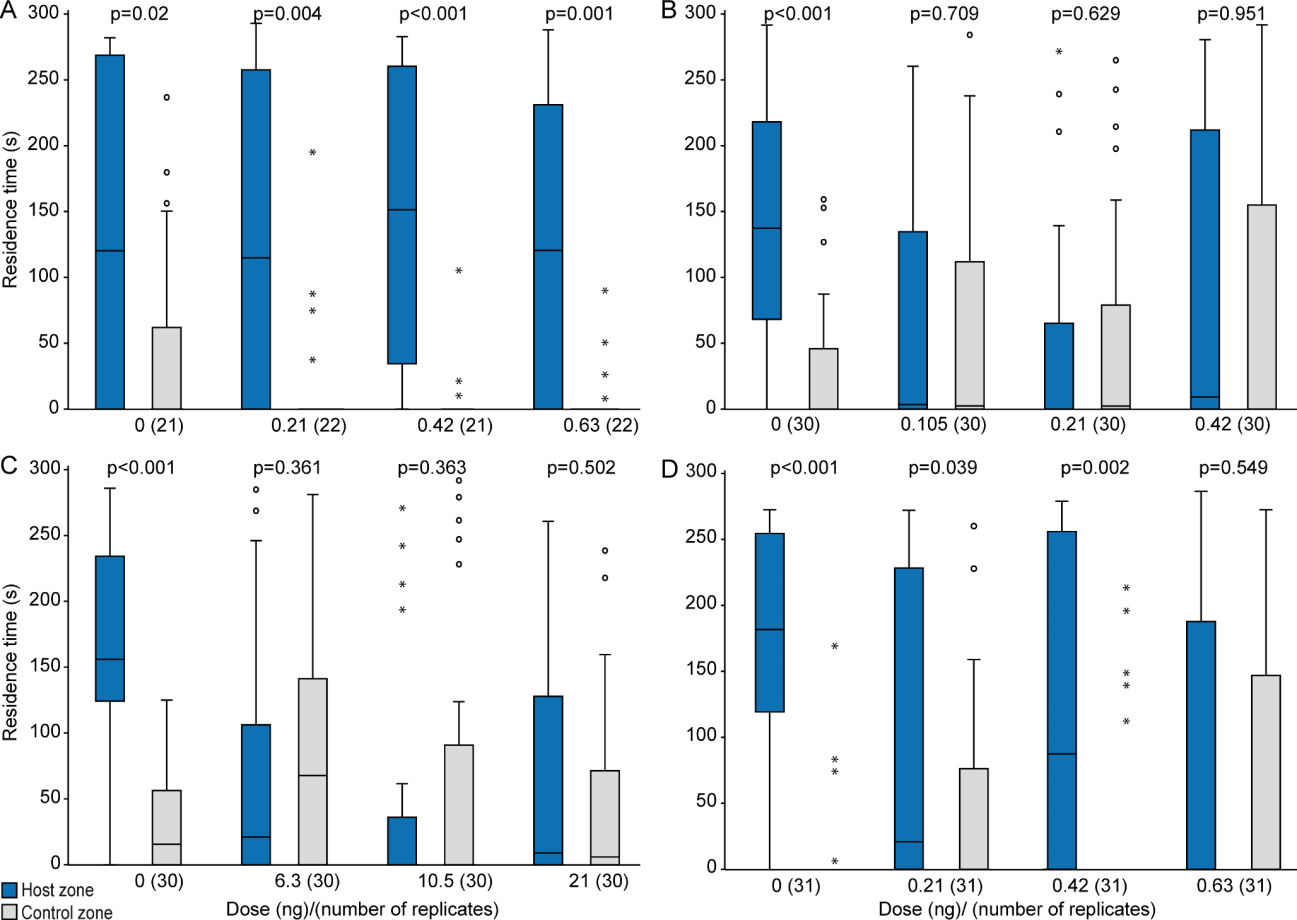
Effect of various doses of **A**. acetamiprid, **B**. dimethoate, **C**. flupyradifurone, or **D**. sulfoxaflor on the response of experienced *Leptopilina heterotoma* females to host odor in a T-olfactometer. Shown are the residence times of females in the host zone (*Drosophila-*medium with 5 host larvae) and the untreated control zone of the olfactometer. Control females were treated with pure acetone (dose 0 ng). Box-and-whisker plots show 25 and 75% quartiles (upper and lower end of the boxes), median (horizontal line in between), 1.5× the interquartile range (whiskers), and outliers (° means > 1.5 × and * means > 3 × interquartile range). The p-values are based on comparison between treatments and the respective control (Wilcoxon matched pairs test; number of replicates given in brackets)

*Effects on Attraction of Females to Host Odor.* In all control experiments, acetone-treated females showed a significant preference for the host odor (Fig. 5A-D). Acetamiprid had no effect on the females’ preference at the tested dose range. In contrast, all doses of dimethoate (≥ 0.105 ng) and flupyradifurone (≥ 6.3ng) as well as the highest dose of sulfoxaflor (0.63 ng) disrupted the orientation of females towards host odor.

### Transfer of Insecticides via The Host

**Fig. 6:**
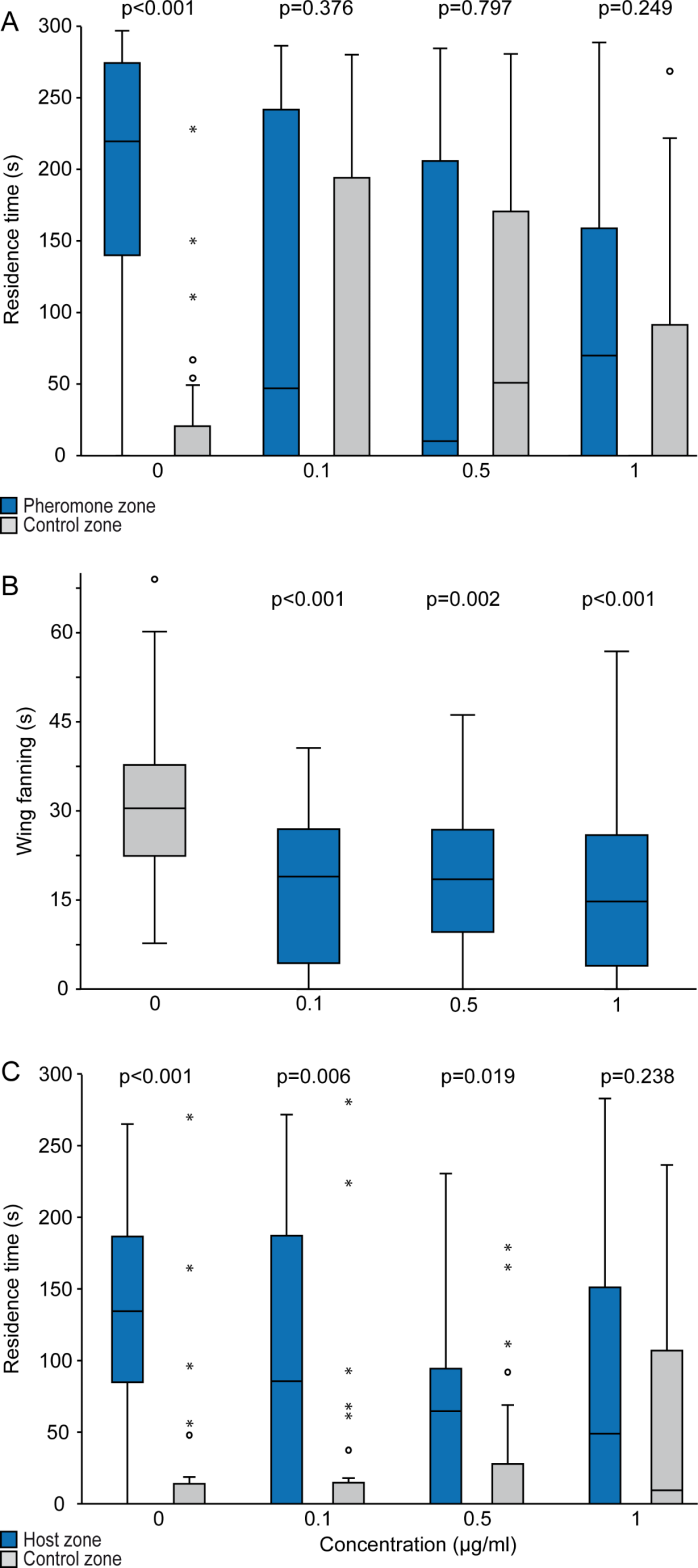
Effects of dimethoate on pheromone communication and host-finding of *Leptopilina heterotoma* after uptake of the active substance via the host. Hosts (*Drosophila melanogaster* larvae) were reared on rearing medium (1.6 g instant medium + 10 ml water) supplemented with 1 ml of a dimethoate solution (in 10% acetone in water, concentration given on the x-axis) **A**. Response of males towards female-derived pheromone extract in a T-olfactometer. Shown are the residence times of males in the pheromone zone and the solvent-treated control zone of the olfactometer. **B**. Wing-fanning duration of males towards female-derived pheromone extract within a 5 min observation period. **C**. Response of experienced females to host odor in a T-olfactometer. Shown are the residence times of females in the host zone (*Drosophila-*medium with 5 host larvae) and the untreated control zone of the olfactometer. Box-and-whisker plots show 25 and 75% quartiles (upper and lower end of the boxes), median (horizontal line in between), 1.5× the interquartile range (whiskers), and outliers (° means > 1.5 × and * means > 3 × interquartile range). The p-values are based on comparison between treatments and the respective control; (**A**, **C**) Wilcoxon matched pairs test, (**B**) Kruskal-Wallis test followed by Dunn’s test (*n* = 30)

Male and female *L. heterotoma* developing on hosts reared on the control DFM showed significant preferences for the pheromone and host zone, respectively (Fig. 6A, C). Males no longer preferred the pheromone in the T-olfactometer when developing on hosts reared on dimethoate-treated DFM at any tested concentration (Fig. 5a) and also the duration of wing fanning towards pheromone-treated paper disks was significantly reduced (Fig. 5B). The preference of females for host odor was no longer present when developing on hosts reared on DFM treated with 1 µg/ml dimethoate.

## Discussion

Proper olfactory system function is a key factor in the ability of parasitic wasps to mate (Ruther 2013) as well as locate their hosts (Turlings and Erb 2018; Wäschke et al. 2013). Doses of all four insecticides affected at least one of the three parameters tested in this study acting as proxies for partner and host finding abilities with first effects being seen at sub-nanogram (acetamiprid, dimethoate, and sulfoxaflor) and nanogram levels (flupyradifurone), respectively (Tab. S3 in the Supplementary Information). At the lowest bioactive doses, > 90% of the treated wasps survive the insecticide treatment (Table 1). Furthermore, pheromone recognition and host finding were also disturbed in those *L. heterotoma* that had developed in *D. melanogaster* larvae reared on dimethoate-treated DFM at sublethal levels. Hence, doses of this insecticide sufficient to interfere with chemical orientation of *L. heterotoma* can reach the parasitic wasp via the food chain.

Male *L. heterotoma* differ from some congeneric species in that they disperse from their natal patch faster after emergence and therefore depend on the volatile iridoids to locate females over longer distances (Böttinger and Stökl 2020). By doing so, males that are able to mate with females from different natal patches avoid the risk of inbreeding. Given that the males’ response to the female sex pheromone is impacted even by the lowest insecticide doses, their chance of finding unrelated receptive females will decrease. Males’ pheromone anosmia increases the likelihood of females remaining unmated thus resulting in suboptimal male-biased sex ratios because of the haplodiploid sex determination in parasitic wasps (Gardner 2014).

Also, the lessened or even absent wing fanning response of insecticide-treated *L. heterotoma* males likely translates into decreased mating opportunities. A recent study (Lang et al. 2024) demonstrated that sexual communication in *L. heterotoma* is multimodal with wing fanning behavior correlating with the males’ mating success. Decisive, however, was wing fanning frequency and not its duration. Hence vibrometric studies are necessary to investigate whether not only the duration but also the frequency of wing fanning is negatively impacted by insecticide treatment. As wing-fanning is a result of muscle contraction, inhibition of proper motor function might also have contributed to the reduced wing fanning response in *L. heterotoma* males. In bees, for example, flupyradifurone has been shown to have effects on the sense of taste, appetitive learning (Hesselbach and Scheiner 2018) and motor function (Hesselbach and Scheiner 2019). Mating experiments with *L. distinguendus* (Schöfer et al. 2024) and *N*. *vitripennis* (Schöfer et al. 2023) revealed that all four insecticides tested in this study reduced the mating frequency at least at one dose. Comparable experiments with *L. heterotoma* were not possible without considerable additional effort, as the mating rate is already very low (< 20%) in untreated wasps (Lang et al. 2024) likely due to female mate choice.

Females of *L. heterotoma* use the major component of their sex pheromone, (-)-iridomyrmecin, also as competition avoidance cue during host finding (Weiss et al. 2013). It will be interesting to study whether this ability is similarly hampered by sublethal insecticide exposure like the male pheromone response. Three of the four tested insecticides interfered within the tested dose range with the females’ host finding ability suggesting that the ecosystem service provided by *L. heterotoma* suffers through exposure of the wasps to the active substances. Interestingly, acetamiprid, while interfering with the pheromone communication of *L. heterotoma* males, had no significant effects on olfactory host finding of the females. Conversely, sulfoxaflor interfered with the females’ host finding ability in *N. vitripennis* but had no significant effects on their response to the male sex pheromone (Schöfer et al. 2023). This suggests that chemical signals used in the context of sexual communication and host finding, respectively, are processed through different neuronal circuits in the brain of parasitic wasps as is well-known, for instance, from Lepidoptera (Renou 2014). Results furthermore suggest that nAChR involved in these circuits differ in their susceptibility to the different receptor agonists. This is in agreement with previous work with the parasitoid *Telenomus busseolae* where even active substances of the same class of insecticides (pyrethroids) impacted olfactory host finding differently. Pyrethroids function by preventing the closure of the voltage-gated sodium channels in the axonal membranes of neurons (Davies et al. 2007). While cyfluthrin disrupted kairomone-mediated host finding at LC_25_, deltamethrin did not (Bayram et al. 2010). In *L. heterotoma*, deltamethrin as well as the organophosphate chlorpyrifos tested at LC_20_ even improved the kairomone response (Delpuech et al. 2005). These results show that sublethal effects of insecticides on insects can be quite variable. We therefore propose that registration procedures of new active substances should investigate several species in different contexts.

While it is reasonable and tempting to assume that the effects observed in this study are caused by direct interactions of nAChR agonists and AChE, respectively, with cholinergic neurons of the olfactory system, latest research suggests that at least the neonicotinoid imidacloprid interferes with host and mate finding of parasitic wasps indirectly by downregulating olfactory receptors (Or) (Shi et al. 2024). Males and females of *Leptopilina drosophilae* fed imidacloprid in sugar solution (LC_10_) showed a decreased host finding ability, and treated males were less successful during courtship and mating. These effects came along with downregulation of some Or genes, and RNAi-mediated knock-down of these genes led to similar effects as imidacloprid treatment (Shi et al. 2024). Another recent study demonstrated that the pyrethroid bifenthrin interferes at sublethal levels with sexual communication and mating success in the moth *Conogethes punctiferalis* by reducing sex pheromone production (An et al. 2024). Hence, other mechanisms than direct interaction of insecticides with neurons’ neurotransmitter receptors should be considered in future research on the sublethal effects of pesticides on beneficial non-target insects.

Comparison of the acute toxicity of the three nAChR agonist insecticides against *L. heterotoma* with data obtained with *L. distinguendus* (Schöfer et al. 2024) and *N. vitripennis* (Schöfer et al. 2023) revealed stark differences with *L. distinguendus* being most susceptible followed by *L. distinguendus* and *N. vitripennis* (Figs. S1 and S2 in the Supplementary Information). In contrast, the AChE inhibitor dimethoate was similarly toxic for the three species. In honey bees dimethoate and other organophosphates are bioactivated by metabolizing enzymes, with their metabolites binding to AChE (Christen et al. 2019). While the targets of these metabolites are generally the same two forms of AChE (Kim and Lee 2013), insecticides targeting nAChR have been shown to interact with discrete receptors and subunits (Moffat et al. 2016), the form and function of which are different between even closely related species. These differences may explain the strongly fluctuating sensitivity of the three species to the three nAChR antagonists, while the sensitivity to dimethoate was similar.

In the present study, insecticides were applied topically as acetone solutions to the abdomen. Topical application is the most common method for insecticide application in toxicological studies with insects (Matsumura 2012). If, like in our study, the insecticide solution is fully absorbed by the insect, it allows exact control of the dose taken up. A more realistic scenario of insecticide exposure, however, is contact with treated plants (Prabhaker et al. 2011) or the consumption of contaminated nectar (Jervis et al. 1993), honeydew (Wäckers et al. 2008), and guttation water (Urbaneja-Bernat et al. 2020) that all can contain considerable amounts of pesticide residues (Calvo-Agudo et al. 2022; Schmolke et al. 2018; Zioga et al. 2020). Acetamiprid, dimethoate and sulfoxaflor, for instance, increased mortality in the parasitoid *Anagyrus vladimiri* feeding on nectar of insecticide-treated buckwheat (Molina et al. 2024). More research is required under both laboratory and field conditions to investigate whether orally acquired insecticides also have effects on olfactory orientation as reported here. The reported amounts of pesticide residues in natural resources exploited by parasitic wasps are highly variable. Considering a feeding volume of approximately 2 µl of artificial nectar (30% glucose solution) that has been estimated for parasitoids of comparable size (Schöfer et al. 2023, 2024) and the highest amounts of the four insecticides reported in floral and extrafloral nectar, the doses tested in this study can be considered field realistic (Tab. S3 in the Supplementary Information). Additionally, insecticides are often used in combination with other pesticides such as fungicides and herbicides which may enhance insecticide toxicity additively or synergistically (Schuhmann et al. 2022). However, our understanding of such effects is still rather incomplete and deserves greater consideration in future studies (Tosi et al. 2022).

A hitherto widely underestimated way of insecticide exposure of parasitic wasps demonstrated here is via the development in hosts containing sublethal amounts of insecticides. The *Drosophila-Leptopilina* model system allows to study this interaction under well-controlled lab conditions. As an oligophagous parasitoid of *Drosophila*, *L. heterotoma* can adapt to numerous environments, usually following populations of potential host species (Quicray et al. 2023). Many *Drosophila* species occur in agricultural ecosystems such as fruit orchards (Deconninck et al. 2024), where pesticide use including the insecticides tested here is common practice (Biddinger et al. 2013; Heller et al. 2020; Schoevaerts et al. 2015). Therefore, insecticide exposure via the food chain is not unrealistic for the species investigated here and even more for congeneric species parasitizing fruit pest like *D. suzukii* (Asplen et al. 2015). *Leptopilina japonica*, for instance, develops successfully in *D. suzukii* (Asplen et al. 2015) and, like *L. heterotoma*, uses iridoids for sexual communication (Böttinger et al. 2019). Therefore, also other *Drosophila* parasitoids being more relevant than *L. heterotoma* as natural enemies of agricultural pests could be affected by sublethal effects of insecticides in a similar way. However, more studies are necessary investigating typical plant-herbivore-parasitoid model systems under field-realistic conditions to conclude whether the ecosystem service provided by parasitic wasps as natural enemies is compromised largely unnoticedly by anosmia due to sublethal insecticide-exposure via the food chain.

## Electronic Supplementary Material

Below is the link to the electronic supplementary material.

Supplementary Material 1

Supplementary Material 2

Supplementary Material 3

Supplementary Material 4

## Data Availability

All data are given in the supporting information.

## References

[CR1] AAT Bioquest I (2023) Quest Graph™ LD50 calculator. AAT Bioquest, Inc, Sunnyvale, California

[CR2] Agathokleous E, Blande JD, Calabrese EJ, Guedes RNC, Benelli G (2023a) Stimulation of insect vectors of pathogens by sublethal environmental contaminants: a hidden threat to human and environmental health? Environ Poll 336:122422. 10.1016/j.envpol.2023.122422 10.1016/j.envpol.2023.122422 37604394

[CR3] Agathokleous E et al (2023b) Sublethal chemical stimulation of arthropod parasitoids and parasites of agricultural and environmental importance. Environ Res 237. 10.1016/j.envres.2023.116876 10.1016/j.envres.2023.116876 37573021

[CR4] Ahmad S et al (2022) Dimethoate residues in Pakistan and mitigation strategies through microbial degradation: a review. Environ Sci Pollut Res 29:51367–51383. 10.1007/s11356-022-20933-4 10.1007/s11356-022-20933-4 35616845

[CR5] An E, Zhang Y, Yao S (2024) Bifenthrin at sublethal concentrations suppresses mating and laying of female *Conogethes punctiferalis* by regulating sex pheromone biosynthesis and JH signals. J Agric Food Chem 72:22908–22917. 10.1021/acs.jafc.4c06445 39365739 10.1021/acs.jafc.4c06445 PMC11487570

[CR6] Asplen MK et al (2015) Invasion biology of spotted wing Drosophila (*Drosophila suzukii*): a global perspective and future priorities. J Pest Sci 88:469–494. 10.1007/s10340-015-0681-z

[CR7] Barrett GW, Darnell RM (1967) Effects of dimethoate on small mammal populations. Am Midl Nat 77:164–175. 10.2307/2423436

[CR8] Bartlett AJ et al (2018) Lethal and sublethal toxicity of neonicotinoid and butenolide insecticides to the mayfly, *Hexagenia* spp. Environ Poll 238:63–75. 10.1016/j.envpol.2018.03.004 10.1016/j.envpol.2018.03.004 29544197

[CR9] Bass C, Denholm I, Williamson MS, Nauen R (2015) The global status of insect resistance to neonicotinoid insecticides. Pest Biochem Physiol 121:78–87. 10.1016/j.pestbp.2015.04.004 10.1016/j.pestbp.2015.04.004 26047114

[CR10] Bayram A, Salerno G, Onofri A, Conti E (2010) Sub-lethal effects of two pyrethroids on biological parameters and behavioral responses to host cues in the egg parasitoid *Telenomus busseolae*. Biol Control 53:153–160. 10.1016/j.biocontrol.2009.09.012

[CR11] Biddinger DJ et al (2013) Comparative toxicities and synergism of apple orchard pesticides to *Apis mellifera* (L.) and *Osmia cornifrons* (Radoszkowski. PLoS ONE 8:e72587. 10.1371/journal.pone.0072587 24039783 10.1371/journal.pone.0072587 PMC3767698

[CR12] Bohbot JD, Pitts RJ (2015) The narrowing olfactory landscape of insect odorant receptors. Front Ecol Evol 3:39. 10.3389/fevo.2015.00039

[CR14] Böttinger LC, Stökl J (2020) Dispersal from natal patch correlates with the volatility of female sex pheromones in parasitoid wasps. Front Ecol Evol 8. 10.3389/fevo.2020.557527

[CR13] Böttinger LC, Hofferberth J, Ruther J, Stökl J (2019) Semiochemicals mediating defense, intraspecific competition, and mate finding in *Leptopilina ryukyuensis* and *L. japonica* (Hymenoptera: Figitidae), parasitoids of Drosophila. J Chem Ecol:241–252 10.1007/s10886-019-01052-w 10.1007/s10886-019-01052-w 30756216

[CR15] Calvo-Agudo M, Gonzalez-Cabrera J, Pico Y, Calatayud-Vernich P, Urbaneja A, Dicke M, Tena A (2019) Neonicotinoids in excretion product of phloem-feeding insects kill beneficial insects. Proc Natl Acad Sci USA 116:16817–16822. 10.1073/pnas.1904298116 31383752 10.1073/pnas.1904298116 PMC6708310

[CR16] Calvo-Agudo M, Tooker JF, Dicke M, Tena A (2022) Insecticide-contaminated honeydew: risks for beneficial insects. Biol Rev 97:664–678. 10.1111/brv.12817 34802185 10.1111/brv.12817 PMC9299500

[CR17] Chen Y et al (2019) Ecological risk assessment of the increasing use of the neonicotinoid insecticides along the east coast of China. Environ Int 127:550–557. 10.1016/j.envint.2019.04.010 30981913 10.1016/j.envint.2019.04.010

[CR18] Christen V, Joho Y, Vogel M, Fent K (2019) Transcriptional and physiological effects of the pyrethroid deltamethrin and the organophosphate dimethoate in the brain of honey bees (*Apis mellifera*). Environ Poll 244:247–256. 10.1016/j.envpol.2018.10.030 10.1016/j.envpol.2018.10.030 30340169

[CR19] Dáder B, Viñuela E, Moreno A, Plaza M, Garzo E, del Estal P, Fereres A (2019) Sulfoxaflor and natural pyrethrin with piperonyl butoxide are effective alternatives to neonicotinoids against juveniles of Philaenus spumarius, the European vector of Xylella fastidiosa. Insects 10:225. 10.3390/insects10080225 10.3390/insects10080225 PMC6723376 31366061

[CR20] Davies TGE, Field LM, Usherwood PNR, Williamson MS (2007) A comparative study of voltage-gated sodium channels in the Insecta: implications for pyrethroid resistance in anopheline and other neopteran species. Insect Mol Biol 16:361–375. 10.1111/j.1365-2583.2007.00733.x 17433068 10.1111/j.1365-2583.2007.00733.x

[CR21] Deconninck G et al (2024) Fallen fruit: a backup resource during winter shaping fruit fly communities. Agric for Entomol 26:232–248. 10.1111/afe.12610

[CR22] Decourtye A, Devillers J (2010) Ecotoxicity of neonicotinoid insecticides to bees. In: Thany SH (ed) Insect nicotinic acetylcholine receptors. Advances in Experimental Medicine and Biology, vol 683. Springer, New York, pp 85–95. 10.1007/978-1-4419-6445-8 10.1007/978-1-4419-6445-8_8 20737791

[CR24] Delpuech JM, Froment B, Fouillet P, Pompanon F, Janillon S, Bouletreau M (1998a) Inhibition of sex pheromone communications of *Trichogramma brassicae* (Hymenoptera) by the insecticide chlorpyrifos. Environ Toxicol Chem 17:1107–1113. 10.1002/etc.5620170617

[CR25] Delpuech JM, Gareau E, Terrier O, Fouillet P (1998b) Sublethal effects of the insecticide chlorpyrifos on the sex pheromonal communication of *Trichogramma brassicae*. Chemosphere 36:1775–1785. 10.1016/s0045-6535(97)10071-6

[CR23] Delpuech JM, Bardon C, Bouletreau M (2005) Increase of the behavioral response to kairomones by the parasitoid wasp *Leptopilina heterotoma* surviving insecticides Arch. Environ Contam Toxicol 49:186–191. 10.1007/s00244-004-0158-1 10.1007/s00244-004-0158-1 16082580

[CR26] Demirci O, Gungordu A (2020) Evaluation of the biochemical effects of an acetamiprid-based insecticide on a non-target species *Gambusia holbrooki*. Water Environ J 34:481–489. 10.1111/wej.12549

[CR27] Desneux N, Decourtye A, Delpuech JM (2007) The sublethal effects of pesticides on beneficial arthropods. Annu Rev Entomol 52:81–106. 10.1146/annurev.ento.52.110405.091440 16842032 10.1146/annurev.ento.52.110405.091440

[CR28] Dicke M, Van Lenteren JC, Boskamp GJF, van Dongen-van Leeuwen E (1984) Chemical stimuli in host-habitat location by *Leptopilina heterotoma* (Thomson) (Hymenoptera: Eucoilidae), a parasite of *Drosophila*. J Chem Ecol 10:695–712. 10.1007/BF00988537 24318734 10.1007/BF00988537

[CR29] Engenheiro EL, Hankard PK, Sousa JP, Lemos MF, Weeks JM, Soares AMM (2005) Influence of dimethoate on acetylcholinesterase activity and locomotor function in terrestrial isopods. Environ Toxicol Chem 24:603–609. 10.1897/04-131r.1 15779760 10.1897/04-131r.1

[CR30] European Commission (2013) Commission implementing regulation (EU) 485/2013 of 24 May 2013 amending implementing regulation (EU) 540/2011, as regards the conditions of approval of the active substances clothianidin, thiamethoxamand imidacloprid, and prohibiting the use and sale of seeds treated with plant protection products containing those active substances. Off J Eur Union 139:12–26

[CR31] European Food Safety Authority (2016) Peer review of the pesticide risk assessment of the active substance acetamiprid. EFSA J 14:e04610. 10.2903/j.efsa.2016.4610 10.2903/j.efsa.2016.4416 PMC13093159 42016872

[CR32] Exactitude Consultancy (2024) Neonicotinoid pesticide market by type (Imidacloprid, Thiacloprid, Thiamethoxam, Acetamiprid), by crops (cereals, oilseed, pulses, fruits, vegetables and others) and region, global trends and forecast from 2024 to 2030. Accessed 24th of September 2024 https://exactitudeconsultancy.com/reports/38308/neonicotinoid-pesticide-market/

[CR33] Gaboardi SC, Candiotto LZP, Panis C (2023) Agribusiness in Brazil and its dependence on the use of pesticides. Hyg Environ Health Adv 8:100080. 10.1016/j.heha.2023.100080

[CR34] Gardner A (2014) Dynamics of sex ratio and female unmatedness under haplodiploidy. Ecol Evol 4:1623–1628. 10.1002/ece3.1045 24967080 10.1002/ece3.1045 PMC4063463

[CR35] Gill GS, Chong JH (2021) Efficacy of selected insecticides as replacement for neonicotinoids in managing sweetpotato whitefly on Poinsettia. Horttechnology 31:745–752. 10.21273/Horttech04853-21

[CR36] Godfray HCJ (1994) Parasitoids - behavioral and evolutionary ecology. Princeton University Press, Chichester

[CR37] Godfray HCJ et al (2014) A restatement of the natural science evidence base concerning neonicotinoid insecticides and insect pollinators. Proc R Soc B-Biol Sci 281:9. 10.1098/rspb.2014.0558 10.1098/rspb.2014.0558 PMC4046413 24850927

[CR38] Guedes RNC, Smagghe G, Stark JD, Desneux N (2016) Pesticide-induced stress in arthropod pests for optimized integrated pest management programs. Annu Rev Entomol 61:43–62. 10.1146/annurev-ento-010715-023646 26473315 10.1146/annurev-ento-010715-023646

[CR39] Hammer Ø, Harper DA, Ryan PD (2001) PAST: Paleontological statistics software package for education and data analysis Palaeontol Electron 4:9

[CR40] Hedlund K, Vet LEM, Dicke M (1996) Generalist and specialist parasitoid strategies of using odours of adult drosophilid flies when searching for larval hosts. Oikos 77:390–398. 10.2307/3545929

[CR41] Heller S, Joshi NK, Chen J, Rajotte EG, Mullin C, Biddinger DJ (2020) Pollinator exposure to systemic insecticides and fungicides applied in the previous fall and pre-bloom period in apple orchards. Environ Pollut 265. 10.1016/j.envpol.2020.114589 10.1016/j.envpol.2020.114589 32531650

[CR42] Hesselbach H, Scheiner R (2018) Effects of the novel pesticide flupyradifurone (Sivanto) on honeybee taste and cognition. Sci Rep 8. 10.1038/s41598-018-23200-0 10.1038/s41598-018-23200-0 PMC5862975 29563522

[CR43] Hesselbach H, Scheiner R (2019) The novel pesticide flupyradifurone (Sivanto) affects honeybee motor abilities. Ecotoxicology 28:354–366. 10.1007/s10646-019-02028-y 30826953 10.1007/s10646-019-02028-y

[CR44] Horowitz A, Mendelson Z, Weintraub P, Ishaaya I (1998) Comparative toxicity of foliar and systemic applications of acetamiprid and imidacloprid against the cotton whitefly, *Bemisia tabaci* (Hemiptera: Aleyrodidae). Bull Entomol Res 88:437–442. 10.1017/S0007485300042176

[CR45] Jacob CRO, Malaquias JB, Zanardi OZ, Silva CAS, Jacob JFO, Yamamoto PT (2019) Oral acute toxicity and impact of neonicotinoids on *Apis mellifera* L. and *Scaptotrigona postica* Latreille (Hymenoptera: Apidae). Ecotoxicol 28:744–753. 10.1007/s10646-019-02070-w 10.1007/s10646-019-02070-w 31254187

[CR46] Jatsch AS, Ruther J (2021) Acetone application for administration of bioactive substances has no negative effects on longevity, fitness, and sexual communication in a parasitic wasp. PLoS ONE 16:e0245698. 10.1371/journal.pone.0245698 33471848 10.1371/journal.pone.0245698 PMC7816986

[CR47] Jervis MA, Kidd NAC, Fitton MG, Huddleston T, Dawah HA (1993) Flower-visiting by hymenopteran parasitoids. J Nat Hist 27:67–105. 10.1080/00222939300770051

[CR48] Jeschke P, Nauen R (2008) Neonicotinoids - from zero to hero in insecticide chemistry. Pest Manag Sci 64:1084–1098. 10.1002/ps.1631 18712805 10.1002/ps.1631

[CR49] Kim YH, Lee SH (2013) Which acetylcholinesterase functions as the main catalytic enzyme in the Class Insecta? Insect Biochem Mol Biol 43:47–53. 10.1016/j.ibmb.2012.11.004 23168079 10.1016/j.ibmb.2012.11.004

[CR50] Lang SR, Conrad T, Steiger S, Stökl J (2024) Analysing the information content of the multimodal courtship display of a parasitoid wasp. Biol J Linn Soc. 10.1093/biolinnean/blae069

[CR51] Lee JC, Heimpel GE, Leibee GL (2004) Comparing floral nectar and aphid honeydew diets on the longevity and nutrient levels of a parasitoid wasp. Entomol Exp Appl 111:189–199. 10.1111/j.0013-8703.2004.00165.x

[CR52] Lisi F et al (2023) Sublethal effects of nine insecticides on *Drosophila suzukii* and its major pupal parasitoid *Trichopria drosophilae*. Pest Manag Sci 79:5003–5014. 10.1002/ps.7702 37548138 10.1002/ps.7702

[CR53] Liu F, Zhang X, Gui QQ, Xu QJ (2012) Sublethal effects of four insecticides on anagrus nilaparvatae (Hymenoptera: Mymaridae), an important egg parasitoid of the rice planthopper *Nilaparvata lugens* (Homoptera: Delphacidae). Crop Prot 37:13–19. 10.1016/j.cropro.2012.02.012

[CR54] Matsumura F (2012) General principles of insecticide toxicology. In: Matsumura F (ed) Toxicology of insecticides. Springer US, New York, pp 11–43. 10.1007/978-1-4613-2491-1

[CR55] Moffat C et al (2016) Neonicotinoids target distinct nicotinic acetylcholine receptors and neurons, leading to differential risks to bumblebees. Sci Rep 6:24764. 10.1038/srep24764 27124107 10.1038/srep24764 PMC4849185

[CR56] Molina P, Campos-Rivela JM, Agusti N, Ferrer MTM (2024) Impact of direct and indirect ingestion of six systemic pesticides on the parasitoid *Anagyrus vladimiri*. Crop Prot 182. 10.1016/j.cropro.2024.106746

[CR57] Mortimer NT (2013) Parasitoid wasp virulence: a window into fly immunity. Fly 7:242–248. 10.4161/fly.26484 24088661 10.4161/fly.26484 PMC3896496

[CR58] Mörtl M, Darvas B, Vehovszky Á, Győri J, Székács A (2019) Contamination of the guttation liquid of two common weeds with neonicotinoids from coated maize seeds planted in close proximity. Sci Tot Environ 649:1137–1143. 10.1016/j.scitotenv.2018.08.271 10.1016/j.scitotenv.2018.08.271 30308885

[CR59] Mörtl M, Takacs E, Klatyik S, Szekacs A (2020) Appearance of thiacloprid in the guttation liquid of coated maize seeds. Int J Environ Res Pub He 17:14. 10.3390/ijerph17093290 10.3390/ijerph17093290 PMC7246591 32397272

[CR60] Müller C (2018) Impacts of sublethal insecticide exposure on insects - facts and knowledge gaps. Bas Appl Ecol 30:1–10. 10.1016/j.baae.2018.05.001

[CR61] Nauen R et al (2015) Flupyradifurone: a brief profile of a new butenolide insecticide. Pest Manag Sci 71:850–862. 10.1002/ps.3932 25351824 10.1002/ps.3932 PMC4657471

[CR62] O’Brien R (1963) Mode of action of insecticides, binding of organophosphates to cholinesterases. J Agric Food Chem 11:163–166. 10.1021/jf60126a019

[CR63] Papaj DR, Vet LE (1990) Odor learning and foraging success in the parasitoid *Leptopilina heterotoma*. J Chem Ecol 16:3137–3150. 10.1007/BF00979616 24263300 10.1007/BF00979616

[CR64] Peters RS et al (2017) Evolutionary history of the Hymenoptera. Curr Biol 27:1013–1018. 10.1016/j.cub.2017.01.027 28343967 10.1016/j.cub.2017.01.027

[CR65] Pisa LW et al (2015) Effects of neonicotinoids and fipronil on non-target invertebrates. Environ Sci Pollut Res 22:68–102. 10.1007/s11356-014-3471-x 10.1007/s11356-014-3471-x PMC4284392 25223353

[CR66] Prabhaker N, Castle SJ, Naranjo SE, Toscano NC, Morse JG (2011) Compatibility of two systemic neonicotinoids, imidacloprid and thiamethoxam, with various natural enemies of agricultural pests. J Econ Entomol 104:773–781. 10.1603/ec10362 21735893 10.1603/ec10362

[CR67] Quicke DLJ (1997) Parasitic wasps. Chapman & Hall, London

[CR68] Quicray M, Wilhelm L, Enriquez T, He SL, Scheifler M, Visser B (2023) The *Drosophila*-parasitizing wasp *Leptopilina heterotoma*: a comprehensive model system. Ecol Evol Ecol Evol 13. 10.1002/ece3.9625 10.1002/ece3.9625 PMC9871341 36703713

[CR69] Rafalimanana H, Kaiser L, Delpuech J-M (2002) Stimulating effects of the insecticide chlorpyrifos on host searching and infestation efficacy of a parasitoid wasp. Pest Manag Sci 58:321–328. 10.1002/ps.454 11975179 10.1002/ps.454

[CR70] Renou M (2014) Pheromones and general odor perception in insects. In: Mucignat-Caretta C (ed) Neurobiology of Chemical Communication. Taylor & Francis, Boca-Raton (FL), pp 23–56. 10.1201/b16511 24830044

[CR71] Ruther J (2013) Novel insights into pheromone-mediated communication in parasitic hymenopterans. In: Wajnberg E, Colazza S (eds) Chemical ecology of insect parasitoids. Wiley-Blackwell, Chichester, U.K., pp 112–143. 10.1002/9781118409589.ch6

[CR72] Schmolke A, Kearns B, O’Neill B (2018) Plant guttation water as a potential route for pesticide exposure in honey bees: a review of recent literature. Apidologie 49:637–646. 10.1007/s13592-018-0591-1

[CR73] Schoevaerts C, Bangels E, Van Waetermeulen X, Belien T, Haas M, Maeyer L, Petre R (2015) Complementary strategy based on flupyradifurone (sivanto prime^®^) and Spirotetramat (Movento^®^) for integrated *Cacopsylla pyri* control in IPM pears with focus on the temporal discrimination towards beneficials. Acta Hortic 1094:463–470. 10.17660/ActaHortic.2015.1094.61

[CR74] Schöfer N, Ackermann J, Hoheneder J, Hofferberth J, Ruther J (2023) Sublethal effects of four insecticides targeting cholinergic neurons on partner and host finding in the parasitic wasp *Nasonia vitripennis*. Environ Toxicol Chem 42:2400–2411. 10.1002/etc.5721 10.1002/etc.5721 37477474

[CR75] Schöfer N, Ratschmann G, Ruther J (2024) Effects of sub-nanogram doses of acetamiprid, dimethoate, flupyradifurone, and sulfoxaflor on courtship, mating, and olfactory host finding of the parasitic wasp *Lariophagus distinguendus*. Entomol Exp Appl 172:666–678. 10.1111/eea.13444

[CR76] Schuhmann A, Schmid AP, Manzer S, Schulte J, Scheiner R (2022) Interaction of insecticides and fungicides in bees. Front Insect Sci 1:808335. 10.3389/finsc.2021.808335 10.3389/finsc.2021.808335 PMC10926390 38468891

[CR77] Shi WQ et al (2024) Neonicotinoid insecticide imidacloprid induces chemosensory deficits in a nontarget parasitoid wasp. Sci Tot Environ 907. 10.1016/j.scitotenv.2023.168089 10.1016/j.scitotenv.2023.168089 37879478

[CR78] Simon-Delso N et al (2015) Systemic insecticides (neonicotinoids and fipronil): trends, uses, mode of action and metabolites Environ. Sci Pollut Res 22:5–34. 10.1007/s11356-014-3470-y 10.1007/s11356-014-3470-y PMC4284386 25233913

[CR79] Siviter H, Muth F (2020) Do novel insecticides pose a threat to beneficial insects? Proc R Soc B-Biol Sci 287:20201265. 10.1098/rspb.2020.1265 10.1098/rspb.2020.1265 PMC7542824 32993471

[CR80] Sparks TC, Watson GB, Loso MR, Geng C, Babcock JM, Thomas JD (2013) Sulfoxaflor and the sulfoximine insecticides: Chemistry, mode of action and basis for efficacy on resistant insects. Pest Biochem Physiol 107:1–7. 10.1016/j.pestbp.2013.05.014 10.1016/j.pestbp.2013.05.014 25149228

[CR81] Stapel JO, Cortesero AM, Lewis WJ (2000) Disruptive sublethal effects of insecticides on biological control: altered foraging ability and life span of a parasitoid after feeding on extrafloral nectar of cotton treated with systemic insecticides. Biol Control 17:243–249. 10.1006/bcon.1999.0795

[CR82] Steidle JLM, van Loon JJA (2002) Chemoecology of parasitoid and predator oviposition behaviour. In: Hilker M, Meiners T (eds) Chemoecology of Insect Eggs and Egg Deposition. Blackwell, Berlin, pp 291–317. 10.1002/9780470760253.ch11

[CR83] Stökl J, Herzner G (2016) Morphology and ultrastructure of the allomone and sex-pheromone producing mandibular gland of the parasitoid wasp *Leptopilina heterotoma* (Hymenoptera: Figitidae). Arthropod Struct Dev 45:333–340. 10.1016/j.asd.2016.06.003 27349419 10.1016/j.asd.2016.06.003

[CR84] Stökl J, Hofferberth J, Pritschet M, Brummer M, Ruther J (2012) Stereoselective chemical defense in the *Drosophila* parasitoid *Leptopilina heterotoma* is mediated by (–)-iridomyrmecin and (+)-isoiridomyrmecin. J Chem Ecol 38:331–339. 10.1007/s10886-012-0103-0 22477024 10.1007/s10886-012-0103-0

[CR85] Tappert L, Pokorny T, Hofferberth J, Ruther J (2017) Sublethal doses of imidacloprid disrupt sexual communication and host finding in a parasitoid wasp. Sci Rep 7:42756. 10.1038/srep42756 28198464 10.1038/srep42756 PMC5309895

[CR86] Tosi S, Sfeir C, Carnesecchi E, vanEngelsdorp D, Chauzat MP (2022) Lethal, sublethal, and combined effects of pesticides on bees: a meta-analysis and new risk assessment tools. Sci Tot Environ 844:156857. 10.1016/j.scitotenv.2022.156857 10.1016/j.scitotenv.2022.156857 35760183

[CR87] Turlings TCJ, Erb M (2018) Tritrophic interactions mediated by herbivore-induced plant volatiles: mechanisms, ecological relevance, and application potential. Annu Rev Entomol 63:433–452. 10.1146/annurev-ento-020117-043507 29324043 10.1146/annurev-ento-020117-043507

[CR88] Urbaneja-Bernat P, Tena A, González-Cabrera J, Rodriguez-Saona C (2020) Plant guttation provides nutrient-rich food for insects. Proc R Soc B-Biol Sci 287:20201080. 10.1098/rspb.2020.1080 10.1098/rspb.2020.1080 PMC7542811 32933440

[CR89] Wäckers FL, van Rijn PCJ, Heimpel GE (2008) Honeydew as a food source for natural enemies: making the best of a bad meal? Biol Control 45:176–184. 10.1016/j.biocontrol.2008.01.007

[CR90] Wäschke N, Meiners T, Rostas M (2013) Foraging strategies of parasitoids in complex chemical environments. In: Wajnberg E, Colazza S (eds) Chemical ecology of insect parasitoids. Wiley-Blackwell, Chichester, U.K., pp 37–63. 10.1002/9781118409589.ch3

[CR91] Watson GB et al (2011) Novel nicotinic action of the sulfoximine insecticide sulfoxaflor. Insect Biochem Mol Biol 41:432–439. 10.1016/j.ibmb.2011.01.009 21296156 10.1016/j.ibmb.2011.01.009

[CR92] Weiss I, Roessler T, Hofferberth J, Brummer M, Ruther J, Stökl J (2013) A nonspecific defensive compound evolves into a competition avoidance cue and a female sex pheromone. Nat Commun 4:2767. 10.1038/ncomms3767 24231727 10.1038/ncomms3767 PMC3868268

[CR93] Wertheim B, Vet LE, Dicke M (2003) Increased risk of parasitism as ecological costs of using aggregation pheromones: laboratory and field study of *Drosophila-Leptopilina* interaction. Oikos 100:269–282. 10.1034/j.1600-0706.2003.11579.x

[CR94] Wilson RI, Mainen ZF (2006) Early events in olfactory processing. Annu Rev Neurosci 29:163–201. 10.1146/annurev.neuro.29.051605.112950 16776583 10.1146/annurev.neuro.29.051605.112950

[CR95] Zhou HX et al (2022) Comparing the contents, functions and neonicotinoid take-up between floral and extrafloral nectar within a single species (*Hemerocallis citrina* Baroni). Ann Bot 129:429–441. 10.1093/aob/mcac002 35018412 10.1093/aob/mcac002 PMC8944713

[CR96] Zioga E, Kelly R, White B, Stout JC (2020) Plant protection product residues in plant pollen and nectar: a review of current knowledge. Environ Res 189:16. 10.1016/j.envres.2020.109873 10.1016/j.envres.2020.109873 32795671

