# Supplementary material for: Four Neurotoxic Insecticides Impair Partner and Host Finding in the Parasitoid Leptopilina heterotoma and Bioactive Doses Can Be Taken up Via the Host
Source: J Chem Ecol. 2025 Jan 29;51(1):14. doi: 10.1007/s10886-025-01554-w (PMC11779754; doi:10.1007/s10886-025-01554-w)
Supplement: Supplementary file 4 — Supplementary Material 4 [file 10886_2025_1554_MOESM4_ESM.pdf]

Supporting Information

Four Neurotoxic Insecticides Impair Partner and Host Finding in the Parasitoid *Leptopilina heterotoma* and Bioactive Doses Can Be Taken Up Via the Host

NILS SCHÖFER, NATHALIE SAXINGER, KATRIN BRAUMANDL

AND JOACHIM RUTHER*

University of Regensburg, Institute of Zoology, Universitätsstraße 31, 93053 Regensburg, Germany

Table S1 Results of the toxicity tests with *Leptopilina heterotoma* treated with 210 nl each of acetone solutions of (a) acetamiprid, (b) dimethoate, (c) flupyradifurone, and (d) sulfoxaflor. Given is the number of live/dead wasps (evaluated 72 h after the treatment). Statistical analysis by Fisher's exact test, p-values refer to the comparison between pesticide doses and the respective controls (0 ng, pure acetone).

a) Acetamiprid

Dose (ng)	21	10.5	6.3	4.2	2.1	1.05	0.63	0
Rep. 1	0/8	1/7	3/5	4/4	4/4	7/1	7/1	8/0
Rep. 2	0/8	0/8	5/3	3/5	3/5	7/1	7/1	8/0
Rep. 3	1/7	2/6	2/6	4/4	5/3	3/5	4/4	7/1
Rep. 4	1/7	1/7	2/6	6/2	4/4	6/2	8/0	7/1
Rep. 5	0/8	0/8	1/7	2/6	3/5	5/3	5/3	7/1
Rep. 6	1/7	1/7	2/6	3/5	5/3	6/2	8/0	8/0
**total**	**3/45**	**5/43**	**15/33**	**22/26**	**24/24**	**34/14**	**39/9**	**45/3**
p-value	<0.001	<0.001	<0.001	<0.001	<0.001	0.006	0.120	

b) Dimethoate

Dose (ng)	21	10.5	6.3	4.2	2.1	1.05	0.63	0
Rep. 1	0/8	1/7	0/8	0/8	3/5	4/4	5/3	7/1
Rep. 2	0/8	3/5	4/4	7/1	2/6	4/4	6/2	8/0
Rep. 3	0/8	0/8	0/8	0/8	0/8	4/4	4/4	8/0
Rep. 4	0/8	0/8	0/8	1/7	1/7	4/4	8/0	8/0
Rep. 5	0/8	0/8	0/8	0/8	1/7	3/5	4/4	7/1
Rep. 6	0/8	2/6	1/7	2/6	8/0	8/0	8/0	8/0
**total**	**0/48**	**6/42**	**5/43**	**10/38**	**15/33**	**27/21**	**35/13**	**46/2**
p-value	<0.001	<0.001	<0.001	<0.001	<0.001	<0.001	0.0037	

c) Flupyradifurone

Dose (ng)	210	105	21	10.5	6.3	2.1	1.05	0.63	0.21	0
Rep. 1	0/8	1/7	6/2	7/1	6/2	6/2	7/1	8/0	7/1	8/0
Rep. 2	3/5	3/5	8/0	8/0	8/0	7/1	7/1	7/1	8/0	8/0
Rep. 3	0/8	0/8	7/1	7/1	7/1	7/1	8/0	8/0	6/2	8/0
Rep. 4	0/8	1/7	7/1	7/1	7/1	7/1	8/0	8/0	7/1	8/0
Rep. 5	1/7	1/7	3/5	4/4	5/3	7/1	6/2	7/1	7/1	8/0
Rep. 6	0/8	0/8	5/3	3/5	5/3	7/1	7/1	6/2	8/0	6/2
**total**	**4/44**	**6/42**	**36/12**	**36/12**	**38/10**	**41/7**	**43/5**	**44/4**	**43/5**	**46/2**
p-value	<0.001	<0.001	0.007	0.007	0.027	0.159	0.435	0.677	0.435	

d) Sulfoxaflor

Dose (ng)	210	105	21	10.5	6.3	2.1	1.05	0.63	0.42	0.21	0
Rep. 1	0/8	0/8	0/8	0/8	0/8	0/8	4/4	3/5	6/2	7/1	7/1
Rep. 2	0/8	0/8	0/8	0/8	0/8	3/5	6/2	8/0	8/0	6/2	8/0
Rep. 3	0/8	0/8	0/8	0/8	0/8	1/7	2/6	6/2	8/0	8/0	8/0
Rep. 4	0/8	0/8	0/8	0/8	1/7	1/7	7/1	8/0	8/0	8/0	8/0
Rep. 5	0/8	0/8	0/8	0/8	0/8	2/6	5/3	6/2	7/1	7/1	8/0
Rep. 6	0/8	0/8	0/8	0/8	1/7	3/5	4/4	7/1	7/1	8/0	8/0
**total**	**0/48**	**0/48**	**0/48**	**0/48**	**2/46**	**10/38**	**28/20**	**38/10**	**44/4**	**44/4**	**47/1**
p-value	<0.001	<0.001	<0.001	<0.001	<0.001	<0.001	<0.001	0.008	0.362	0.362	

Table S2 Results of the toxicity tests with (a) adults and (b) larvae of *Drosophila melanogaster.* Given is the number of live/dead individuals kept on rearing medium treated with different concentrations of dimethoate. 1 ml of a the dimethoate dilutions (dissolved in 10% acetone/water) were added to the feeding medium (1.6 g instant medium + 10 ml water). Mortality was evaluated after eight days (n=6 with 20 individuals each).

a)

µg/mL	A1	A2	A3	A4	A5	A6	total	% mortality
10000	0/20	0/20	0/20	0/20	0/20	0/20	0/120	100
1000	0/20	0/20	0/20	0/20	0/20	0/20	0/120	100
100	0/20	0/20	0/20	0/20	0/20	0/20	0/120	100
10	0/20	0/20	10/10	0/20	0/20	0/20	10/110	91.7
1.0	20/0	20/0	18/2	14/6	16/4	17/3	105/15	12.5
0.1	20/0	20/0	20/0	20/0	20/0	20/0	120/0	0
0	20/0	20/0	20/0	20/0	17/3	17/3	114/6	5

b)

µg/mL	L1	L2	L3	L4	L5	L6	total	% mortality
10000	0/20	0/20	0/20	0/20	0/20	0/20	0/120	100
1000	0/20	0/20	0/20	0/20	0/20	0/20	0/120	100
100	0/20	0/20	0/20	0/20	0/20	0/20	0/120	100
10	0/20	0/20	0/20	0/20	0/20	0/20	0/120	100
1.0	16/4	14/6	7/13	20/0	17/3	15/5	89/31	25.8
0.1	20/0	20/0	20/20	20/0	20/0	20/0	120/0	0
0	15/5	18/2	19/20	15/5	18/2	19/1	104/16	13.3

Table S3 Literature data on the contamination of floral and extrafloral[a] nectar with the four insecticides tested in this study. Values refer to an assumed consumption of 2 µl by *Leptopilina heterotoma.* For comparison, the lowest sublethal doses are given having shown significant effects in this study in at least one of the bioassays.

	Amount in nectar (ng/2mg ≈ ng/2µl)	First significant effects in this study	Reference
Acetamiprid	0.0001 - 0.0152	0.21	Zioga et al. 2020
	0.14		Heller et al. 2020
	0.012		Azpiazu et al. 2019
	0.02-0.36		Capela et al. 2022
	0.024 / 0.194[a]		Zhou et al. 2022b
	up to 0.026		Pohorecka et al. 2012
	up to 0.012		Demares et al. 2022
	up to 0.035		Cheng et al. 2024
Flupyradifurone	0.52	2.1	Campbell et al. 2016
	8.0		Siviter and Muth 2022
	0.4-3.0 / up to 31.8[a]		EPA 2015
	up to 0.004		Bishop et al. 2020
	up to 1.3		English et al. 2024
Dimethoate	0.2 - 45.9	0.1	Zioga et al. 2020
Sulfoxaflor	0.014 - 0.028	0.21	Jiang et al. 2020
	0.01-0.094		Siviter et al. 2019
	0.4		Zhou et al. 2022a
	0.06 – 2.0		EPA 2016
	0.04-0.14 / 1.0-14.1[a]		Zhou et al. 2023

[a]extrafloral nectar

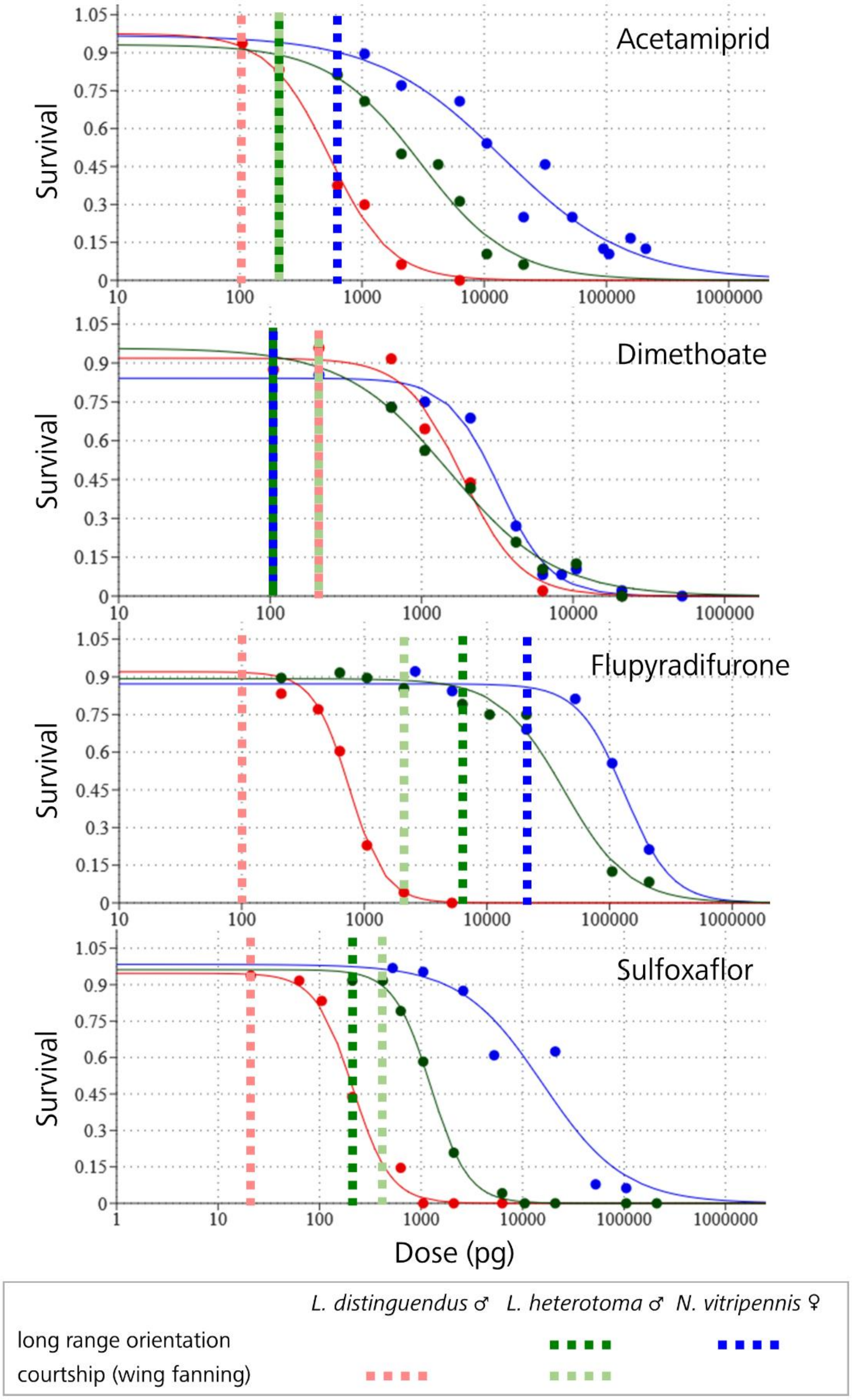

Fig. S1 Relationship between acute toxicity and sublethal effects on pheromone communication in three parasitoid wasp species. Shown are the dose-response curves of the toxicity tests (survival, 72 h after insecticide treatment) of the four active substances in *Lariophagus distinguendus* (red, Schöfer et al. 2024), *Nasonia vitripennis* (blue, Schöfer et al. 2023) and *Leptopilina heterotoma* (green, this study). The lowest dose at which negative effects on pheromone communication occurred in the three species are indicated by dashed lines.

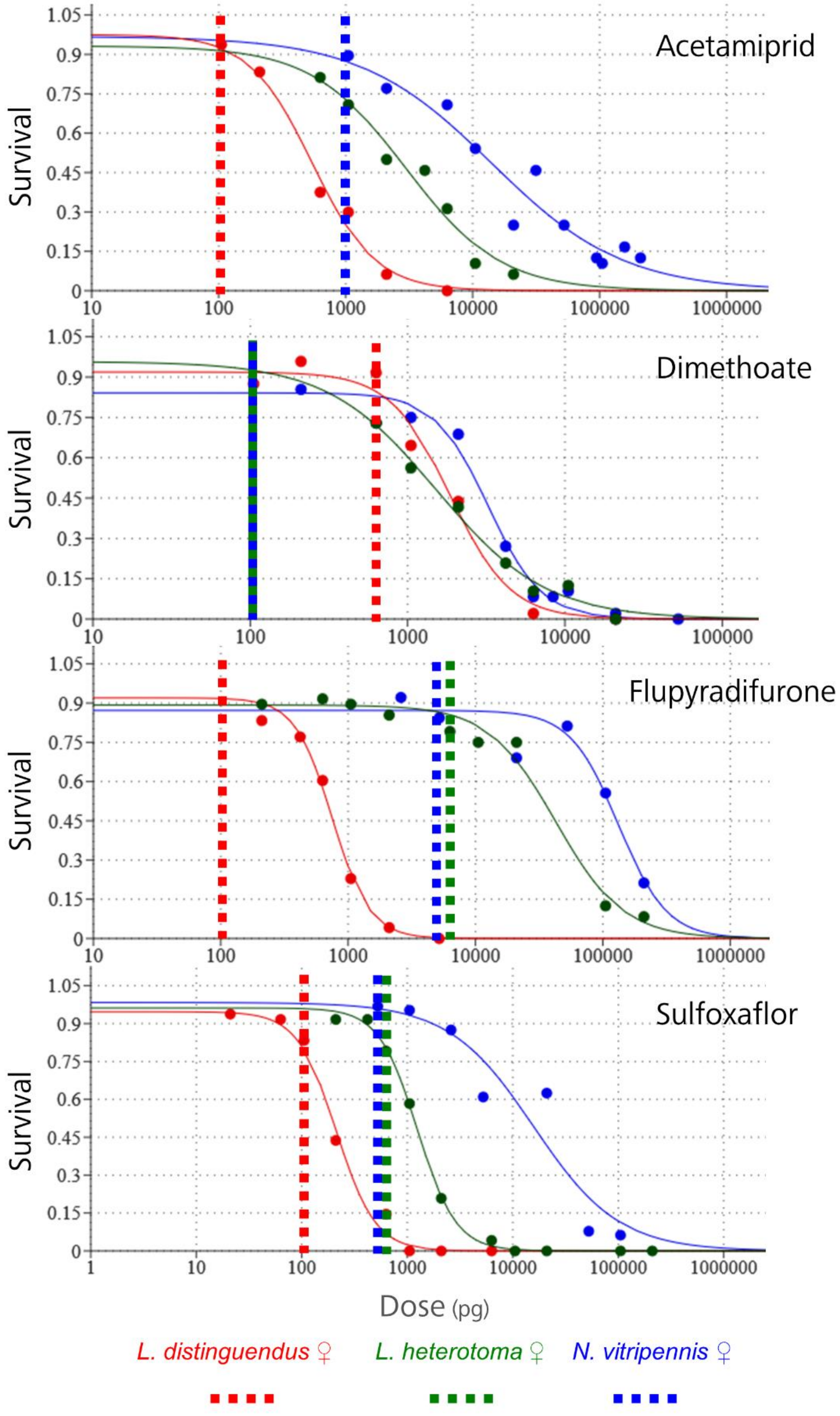

Fig. S2 Relationship between acute toxicity and sublethal effects on olfactory host finding in three parasitoid wasp species. Shown are the dose-response curves of the toxicity tests (survival, 72 h after insecticide treatment) of the four active substances in *Lariophagus distinguendus* (red, Schöfer et al. 2024), *Nasonia vitripennis* (blue, (chöfer et al. 2023) and *Leptopilina heterotoma* (green, this study). The lowest dose at which negative effects on olfactory host finding occurred in the three species are indicated by dashed lines.

REFERENCES

Azpiazu C, Bosch J, Vinuela E, Medrzycki P, Teper D, Sgolastra F (2019) Chronic oral exposure to field-realistic pesticide combinations via pollen and nectar: effects on feeding and thermal performance in a solitary bee Sci Rep 9:11 https://doi.org/10.1038/s41598-019-50255-4

Bishop CA, Woundneh MB, Maisonneuve F, Common J, Elliott JE, Moran AJ (2020) Determination of neonicotinoids and butenolide residues in avian and insect pollinators and their ambient environment in Western Canada (2017, 2018) Sci Tot Environ 737:139386 https://doi.org/10.1016/j.scitotenv.2020.139386

Campbell JW, Cabrera AR, Stanley-Stahr C, Ellis JD (2016) An evaluation of the honey bee (Hymenoptera: Apidae) safety profile of a new systemic insecticide, flupyradifurone, under field conditions in Florida J Econ Entomol 109:1967-1972 https://doi.org/10.1093/jee/tow186

Capela N, Xu M, Simoes S, Azevedo-Pereira H, Peters J, Sousa JP (2022) Exposure and risk assessment of acetamiprid in honey bee colonies under a real exposure scenario in *Eucalyptus* sp. landscapes Sci Tot Environ 840:156485 https://doi.org/10.1016/j.scitotenv.2022.156485

Cheng Q, Zheng S, Wang R, Zeng C, Li K, Lu C (2024) Characterization of near-field temporal and spatial variations of pesticide residues using honeybee specimens as bio-sensing matrices J Environ Manage 365:121598 https://doi.org/10.1016/j.jenvman.2024.121598

Demares FJ et al. (2022) Honey bee (*Apis mellifera*) exposure to pesticide residues in nectar and pollen in urban and suburban environments from four regions of the United States Environ Toxicol Chem 41:991-1003 https://doi.org/10.1002/etc.5298

English SG, Bishop CA, Bieber M, Elliott JE (2024) Following regulation, imidacloprid persists and flupyradifurone increases in nontarget wildlife Environ Toxicol Chem 43:1497-1508 https://doi.org/10.1002/etc.5892

EPA (2015) Environmental fate and ecological risk assessment for foliar, soil drench, and seed treatment uses of the new insecticide flupyradifurone (BYI 02960). Washington, DC: Environmental Protection Agency https://www.regulations.gov/document/EPA-HQ-OPP-2013-0226-0047

EPA (2016) Addendum for the proposed section 3 registration of TransformTM WG and CloserTM SC (Sulfoxaflor) for use on various crops, turf, and ornaments. Washington, DC: Environmental Protection Agency

Heller S, Joshi NK, Chen J, Rajotte EG, Mullin C, Biddinger DJ (2020) Pollinator exposure to systemic insecticides and fungicides applied in the previous fall and pre-bloom period in apple orchards Environ Poll 265:114589 https://doi.org/10.1016/j.envpol.2020.114589

Jiang H, Chen JJ, Zhao C, Tian YQ, Zhang ZX, Xu HH (2020) Sulfoxaflor residues in pollen and nectar of cotton applied through drip irrigation and their potential exposure to *Apis mellifera* L Insects 11:114 https://doi.org/10.3390/insects11020114

Pohorecka K et al. (2012) Residues of neonicotonoid insecticides in bee collected plant material from oilseed rape crops and their effect on bee colonies J Apicul Sci 56:115-134 https://doi.org/10.2478/v10289-012-0029-3

Schöfer N, Ackermann J, Hoheneder J, Hofferberth J, Ruther J (2023) Sublethal effects of four insecticides targeting cholinergic neurons on partner and host finding in the parasitic wasp *Nasonia vitripennis* Environ Toxicol Chem 42:2400-2411 https://doi.org/10.1002/etc.5721

Schöfer N, Ratschmann G, Ruther J (2024) Effects of sub-nanogram doses of acetamiprid, dimethoate, flupyradifurone, and sulfoxaflor on courtship, mating, and olfactory host finding of the parasitic wasp Lariophagus distinguendus Entomol Exp Appl 172:666-678 https://doi.org/10.1111/eea.13444

Siviter H, Muth F (2022) Exposure to the novel insecticide flupyradifurone impairs bumblebee feeding motivation, learning, and memory retention Environ Poll 307:119575 https://doi.org/10.1016/j.envpol.2022.119575

Siviter H, Scott A, Pasquier G, Pull CD, Brown MJF, Leadbeater E (2019) No evidence for negative impacts of acute sulfoxaflor exposure on bee olfactory conditioning or working memory PeerJ 7:22 https://doi.org/10.7717/peerj.7208

Zhou HX et al. (2022a) Identification and quantitation of the novel insecticide sulfoxaflor and its metabolites in floral nectar from Salvia splendens Ker Gawl. (Lamiaceae) Ecotox 31:1310-1320 https://doi.org/10.1007/s10646-022-02590-y

Zhou HX et al. (2023) Residues of sulfoxaflor and its metabolites in floral and extrafloral nectar from *Hibiscus rosa-sinensis* L. (Malvaceae) with or without co-application of tebuconazole Pest Biochem Physiol 196:105587 https://doi.org/10.1016/j.pestbp.2023.105587

Zhou HX et al. (2022b) Comparing the contents, functions and neonicotinoid take-up between floral and extrafloral nectar within a single species (Hemerocallis citrina Baroni) Ann Bot-London 129:429-441 https://doi.org/10.1093/aob/mcac002

Zioga E, Kelly R, White B, Stout JC (2020) Plant protection product residues in plant pollen and nectar: A review of current knowledge Environ Res 189:16 https://doi.org/10.1016/j.envres.2020.109873
